# Efficient Biosynthesis of Theanderose, a Potent Prebiotic, Using Amylosucrase from *Deinococcus deserti*

**DOI:** 10.1021/acs.jafc.4c05763

**Published:** 2024-10-31

**Authors:** Jeon-Uk Kang, Yun-Sang So, Gyungcheon Kim, WonJune Lee, Dong-Ho Seo, Hakdong Shin, Sang-Ho Yoo

**Affiliations:** Department of Food Science and Biotechnology, and Carbohydrate Bioproduct Research Center, Sejong University, 209 Neungdong-ro, Gwangjin-gu, Seoul 05006, Republic of Korea

**Keywords:** amylosucrase, theanderose, *Deinococcus deserti*, fecal fermentation, prebiotic

## Abstract

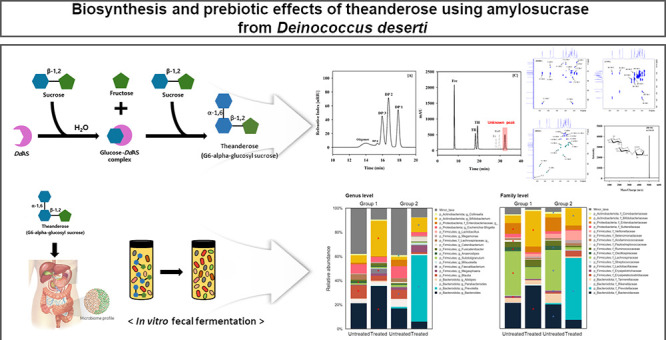

The study aimed to develop an efficient bioprocess for the discovery and synthesis of theanderose by using amylosucrase from *Deinococcus deserti* (*Dd*AS). An unknown trisaccharide produced by *Dd*AS was detected by high-performance anion-exchange chromatography-pulsed amperometric detection and high-performance liquid chromatography-evaporative light scattering detection, purified using medium-pressure liquid chromatography, and identified as theanderose (α-d-glucopyranosyl-(1→6)-α-d-glucopyranosyl-(1→2)-β-d-fructofuranoside) through nuclear magnetic resonance and mass spectrometry. *Dd*AS synthesized theanderose with a 25.4% yield (174.1 g/L) using 2.0 M sucrose at 40 °C for 96 h. In an in vitro digestion model, theanderose showed a 6.5% hydrolysis rate over 16 h. Prebiotic efficacy tests confirmed that theanderose significantly enhanced the proliferation of selected *Bifidobacterium* strains in the culturing medium with theanderose as the main carbon source. Subsequently, fecal fermentation was performed by adding theanderose to the feces of 20 individuals of varying ages to assess its effect on the gut microbiota. Theanderose increased the relative abundance of *Bifidobacteriaceae* and *Prevotellaceae* while decreasing the population ratio of *Lachnospiraceae* and *Ruminococcaceae*. Conclusively, theanderose displayed excellent prebiotic potential when judged by low digestibility and selective growth of beneficial microbes over harmful microbes.

## 1 Introduction

The significance of gut microbiota in human health and disease has spurred interest in the pro-, pre-, and postbiotic modulation of the gut microbiota.^1^ The International Society for the Science of Probiotics and Prebiotics (ISAPP) defines prebiotics as substrates that are selectively used by host microorganisms to offer health benefits.^2^ Common prebiotics, such as inulin, fructooligosaccharides (FOS), and galactooligosaccharides (GOS) facilitate the growth of beneficial gut bacteria (such as *Bifidobacterium* and *Lactobacillus*) and are associated with health benefits, including enhanced gut barrier function, improved insulin sensitivity, and enhanced mineral absorption.^3^ Trisaccharides exhibit distinctive fermentation properties and can selectively nourish certain beneficial gut microorganisms. This contributes to a positive alteration in the gut environment that is characterized by a selective increase in beneficial bacteria.^4,5^ Panose (α-d-glucopyranosyl-(1→6)-α-d-glucopyranosyl-(1→4)-α-d-glucopyranoside), a representative trisaccharide of isomaltooligosaccharides (IMO), facilitates *Bifidobacteria* and *B. lactis* growth and reduced *Bacteroides* and *Clostridium* growth.^6^ Raffinose (α-d-galactosyl-(1→6)-α-d-glucopyranosyl-(1→2)-β-d-fructofuranoside), a trisaccharide observed in legumes, reduced pH in the gut, ammonia concentration, and relative abundance of *Proteobacteria*, while increasing lactate and short-chain fatty acids (SCFAs), total CO_2_ production, and relative abundance of *Bifidobacterium* and *Lactobacillus*.^4,7^ Theanderose (α-d-glucopyranosyl-(1→6)-α-d-glucopyranosyl-(1→2)-β-d-fructofuranose) plays a crucial role in enhancing the freeze resistance of the moss *Physcomitrella patens*.^8^ Additionally, it serves as a natural sweetener with a low caloric value and enhanced taste, making it potentially valuable in the food industry.^9^ However, it is a naturally occurring carbohydrate found in sugar-rich products such as cane sugar and honey. Due to its low concentration (less than 0.3%), large-scale isolation from natural sources is impractical for industrial applications. A previous study reported that theanderose was produced when levansucrase catalyzed a reaction between isomaltose (as an acceptor molecule) and sucrose (as a donor molecule).^9^ Theanderose was poorly degraded by human saliva and porcine pancreas and selectively used by *Bifidobacterium* sp. and *Clostridium butyricum*, which potently contribute to the enhancement of the intestinal environment.^10^

Amylosucrase (ASase; E.C. 2.4.1.4) is a multifunctional enzyme that uses sucrose as the sole substrate to produce glucose, fructose, sucrose isomers (turanose and trehalulose), maltooligosaccharides, and α-1,4-glucan.^11^ ASase is a glycosyltransferase with a high ability to transfer glucose from sucrose to hydroxyl (−OH) groups, efficiently transferring various sugars and natural compounds that possess −OH groups.^12^ ASases have been cloned and characterized from various microbial sources, each displaying variations in ASase reactions.^13^ ASases, which comprise five domains (N, A, B, B’, and C), can produce various proportions of products because of their unique conformations and primary residues within the A, B, and B’ domains that affect specific ASase reactions.^14^ ASase from *Deinococcus geothermalis* (*Dg*AS) produced more trehalulose (1-*O*-α-d-glucopyranosyl-d-fructose) than turanose (α-d-glucopyranosyl-(1→3)-β-d-fructofuranoside) compared to ASase from *Neisseria polysaccharea* (*Np*AS) due to structural differences in their active sites.^15^ Recently, ASase from *Deinococcus deserti* (*Dd*AS) synthesized trehalulose as the major product with yields of up to 246.5 g/L using sucrose (2.0 M) and fructose (0.75 M).^15^ The authors contended that the structural properties of *Dd*AS were more specialized for the production of trehalulose than those of other ASases. Although ASase preferred to attach glucose to glucose through an α-1,4 glycosidic linkage, it could also attach glucose through an α-1,2 or α-1,6 glycosidic linkage, depending on the acceptor and reaction conditions. When *Dg*AS performed a transglycosylation reaction on isoquercitrin (IQ), it transferred glucose to α-1,2-, α-1,4-, and/or α-1,6-glucosidic linkages on the 3-*O*-glucosyl moiety of IQ, depending on the buffer/pH and sucrose concentration.^16^ Additionally, the *Dg*AS-based transglycosylation reaction resulted in the formation of novel isoflavone glycosides with α-glycosidic bonds at the C-7 and/or C-4′ positions of isoflavone aglycones, followed by the production of isoflavone glycosides with α-1,6 glycosidic bonds.^17^ The *Np*AS variants with redesigned +1 and +2 subsites produced the trisaccharides erlose (α-d-glucopyranosyl-(1→4)-α-d-glucopyranosyl-(1→2)-β-d-fructofuranose) and panose using sucrose as the sole substrate.^18^

In this study, *Dd*AS produced significantly higher amounts of trisaccharides compared to *Dg*AS, as determined through high-performance liquid chromatography-evaporative light scattering detection (HPLC-ELSD). This trisaccharide was successfully purified using medium-pressure liquid chromatography (MPLC), and its structure was identified as theanderose using liquid chromatography/mass spectrometry (LC/MS) and nuclear magnetic resonance (NMR). Subsequently, we assessed the biosynthetic optimization of theanderose using *Dd*AS. Additionally, we assessed the growth effects on beneficial bacteria, such as *Bifidobacterium* and *Lactobacillus*, and alterations in gut microbiota through fecal fermentation to confirm the prebiotic effect of theanderose.

## 2 Materials and Methods

### 2.1 Chemicals and Bacterial Strains

Glucose, fructose, sucrose, turanose, trehalulose, maltotriose, and other chemicals were purchased from Sigma-Aldrich (St. Louis, MO, USA) and Merck (Darmstadt, Germany). Porcine pancreatic α-amylase and amyloglucosidase used in the digestion experiments were purchased from Megazyme (Wicklow, Ireland). The probiotic strains used in this study included *Lacticaseibacillus casei* KACC 12413, *Lactobacillus paracasei* KCTC 3510, *Lactobacillus rhamnosus* GG, *Bifidobacterium adolescentis* KCTC 3267, *Bifidobacterium longum* KCTC 3249, and *Bifidobacterium tsurumiense* KACC 16654. The *Escherichia coli* BL21 (DE3) strain harboring the pBT7-N-His-DdAS plasmid was previously constructed in our laboratory.^15,19^

### 2.2 Purification of Recombinant DdAS

Recombinant *E. coli* BL21 (DE3) containing the pBT7-N-His-*Dd*AS plasmid was employed for *Dd*AS expression following a protocol adapted from previous studies.^15^ The cells were cultured in Luria–Bertani (LB) broth with 50 μg/mL ampicillin, maintaining growth at 37 °C until an optical density of 0.6–0.8 at 600 nm was achieved. Protein expression was induced by adding 0.2 mM isopropyl β-d-1-thiogalactopyranoside (IPTG) followed by incubation at 16 °C for 18 h. Cells overexpressing *Dd*AS were harvested through centrifugation (4000 *g*, 4 °C, 10 min), and the pellet was resuspended in 50 mM Tris–HCl buffer (pH 7.0). Cell disruption was achieved using a Vibra Cell VC 750 sonicator (Sonics & Materials, Inc., Newtown, CT) on ice, followed by centrifugation at 8000 *g* for 20 min at 4 °C. The resulting supernatant was passed through a 0.45 μm filter, and His-tagged ASase was isolated via nickel–nitrilotriacetic acid (Ni–NTA) affinity chromatography (QIAGEN, Hilden, Germany). The purified *Dd*AS protein was eluted in buffer containing 50 mM Tris–HCl, pH 7.0, 300 mM NaCl, and 250 mM imidazole, and concentrated using Amicon Ultra-15 centrifugal filters with a 30 K cutoff (Merck Millipore, Carrigtwohill, Ireland). The purity and molecular weight of the *Dd*AS were confirmed by SDS-PAGE analysis (Figure S1). Imidazole was eliminated from the eluted protein fraction via dialysis. Subsequently, the enzyme activity was determined before proceeding with further experimentation.

### 2.3 Determination of Enzyme Activity

*Dd*AS activity was assessed by quantifying the yield of reducing sugars produced through sucrose hydrolysis, according to previous studies.^15^ The assay was performed using a 50 mM sodium phosphate buffer (pH 7.5) supplemented with 100 mM sucrose at 40 °C for 30 min. The reaction was stopped by adding 0.5 mL of 3,5-dinitrosalicylic acid (DNS) solution. Color development occurred by heating the mixture to 100 °C for 5 min, followed by immediate cooling in an ice bath for 5 min to stabilize the chromogenic product. Enzyme activity was determined by constructing a standard curve for fructose concentration (0.01–0.05%), and the absorbance changes were monitored at 575 nm on a DU 730 UV/vis spectrophotometer (Beckman Coulter Inc., Brea, CA, USA). One unit of *Dd*AS activity was defined as the amount of enzyme required to release 1 μmol fructose per minute.

### 2.4 3D Structure Modeling and Molecular Docking

The amino acid homology of *Dd*AS was compared with *Np*AS, *Dg*AS, and *Dr*AS using the NCBI Blast Tool. For *Dd*AS, the amino acid sequence homology was found to be 39.7% for NpAS, 76.3% for DgAS, and 72.4% for *Dr*AS, respectively. The tertiary structure of *Dd*AS used for molecular docking was predicted using the Alphafold2 structure prediction database.^20^ The docking structures of *Dd*AS with sucrose, glucose, and theanderose were then predicted using AutoDock Vina software.^21^ Three independent molecular dynamics (MD) simulations were carried out using the docking structural models of *Dd*AS complexed with theanderose. Simulations were performed with the GROMACS suite (version 2023).^22^ The CHARMM36ff force field was applied to model the protein and theanderose. The systems were solvated in a cubic box of TIP3P water molecules with a minimum distance of 1.0 nm between the protein and the box edges. Na^+^ and Cl^–^ ions were added to neutralize the system and achieve a physiological ion concentration of 0.15 M. Initial structures underwent energy minimization using up to 500,000 steps of steepest descent, followed by 100 ps of NVT equilibration and 100 ps of NPT equilibration with position restraints applied to the protein-heavy atoms. The primary simulations were conducted three times for 60 ns each, utilizing LINCS constraints and a V-rescale thermostat set at 300 K. Root mean square fluctuation (RMSF) were computed for each MD simulation trajectory with the GROMACS suite. The RMSF result from three independent simulations were averaged for *Dd*AS. Plots were created using the Python Matplotlib package (version 3.1.1), and statistical analysis was performed using the Python SciPy package (version 1.3.2).

### 2.5 Assessing Enzyme Reaction Distribution through High-Performance Size Exclusion Chromatography (HPSEC) and HPLC-ELSD

The reaction conditions were set based on the parameters defined in a previous study,^15^ using a 50 mM sodium phosphate buffer (pH 7.5) at 35 °C supplemented with 2.0 M sucrose as the substrate and an enzyme concentration of 400 U/L for 120 h. Following the reaction, the mixture was heat treated in boiling water for 10 min to inactivate the enzyme. Prior to analyzing the reaction product composition via HPSEC and HPLC-ELSD, the sample underwent filtration using a 0.45 μm syringe filter. The filtered sample was analyzed using HPSEC equipped with a refractive index detector and TSK gel G2500PWXL size-exclusion chromatography columns (7.8 mm × 300 mm, 7 μm, TOSOH, Japan) at 78 °C to quantify glucose, maltose, maltotriose, and maltotetraose. A 20 μL injection volume and 100% distilled water as the eluent were used in an isocratic mode at a flow rate of 0.5 mL/min. Additionally, HPLC coupled with ELSD was used for the separation and quantification of theanderose, using a VG-50–4E column (4.6 × 250 mm; Shodex) at 60 °C to quantify glucose, fructose, sucrose, turanose, trehalulose, and theanderose. A 5 μL injection volume, with the mobile phase consisting of methanol:water (1:2) and acetonitrile (ACN) solution was applied in a gradient mode at a flow rate of 1.0 mL/min. The amount of individual sugar components was determined based on the peak area compared with the standard materials. Due to the characteristics of HPLC-ELSD, different types of sugars exhibit varying levels of sensitivity.

### 2.6 Purification of Theanderose

Theanderose was isolated for NMR analysis and straightforward chemical characterization using MPLC. The process used an LC-Forte/R system (YMC Co., Ltd., Kyoto, Japan) that was equipped with a refractive index detector at the Biopolymer Research Center for Advanced Materials at Sejong University. The system employed TSK gel G2500PWXL size-exclusion chromatography columns (21.5 mm × 300 mm, 7 μm; TOSOH, Japan) operating at room temperature. Elution was performed with 100% distilled water at a flow rate of 2.0 mL/min.

### 2.7 Confirmation of the Theanderose Structure

#### 2.7.1 Molecular Weight of Synthesized Theanderose By Liquid Chromatography–Mass Spectrometry (LC-MS) Analysis

The analysis was conducted using an Agilent 6495 Triple quadrupole mass spectrometer (LC-QQQ; Agilent, USA) configured in the negative ionization mode at the Biopolymer Research Center for Advanced Materials at Sejong University. A 5 μL aliquot of the sample was injected into a VG-50–4E column (4.6 × 250 mm; Shodex). The ion spray voltage was adjusted to 4.0 kV, and the temperature was maintained at 350 °C. Nitrogen was used as the nebulizing gas. The mobile phase, consisting of a methanol:water (1:2) and ACN solution, was applied in the gradient mode at a flow rate of 1.0 mL/min. We conducted analyses in both cationic and anionic modes. The intensity values obtained in the cationic mode were consistently lower than those in the anionic mode. Consequently, we focused our analysis on the anionic mode data.

#### 2.7.2 Theanderose Structure Determination Using NMR Analysis

NMR data were acquired at 25 °C (298.15 K) on a Bruker 600 MHz Avance III spectrometer (Bruker Biospin, Rhinstetten Germany) that was equipped with a 5 mm TCI (^1^H/^13^C/^15^N) croy-probe. The ^1^H NMR, ^13^C NMR 1D NMR, and 2D NMR spectra included ^1^H–^1^H correlation spectroscopy (COSY), heteronuclear single quantum correlation (HSQC), and heteronuclear multiple bond correlation (HMBC). Following lyophilization, the purified theanderose (5 mg) was dissolved in pure D_2_O (1.0 mL). Data processing was performed using TopSpin 3.6.4 software (Bruker BioSpin, Rheinstetten, Germany).

### 2.8 Optimization of the Conditions for Theanderose Synthesis

Theanderose synthesis was assessed at various temperatures (30, 35, and 40 °C) in 50 mM sodium phosphate buffer (pH 7.5) and at different pH values (6.0, 7.5, and 9.0) at 40 °C supplemented with 2.0 M sucrose. Before initiating the reaction with 0.3 mg/mL of the *Dd*AS, the substrate solution was preincubated at 40 °C for 10 min. The reaction proceeded for 96 h at 40 °C. Samples were collected at specified intervals, inactivated (boiling for 10 min), and subsequently analyzed. The quantification of theanderose in the soluble reaction products was achieved using high-performance anion-exchange chromatography with pulsed amperometric detection (HPAEC-PAD) on a CarboPac PA1 analytical column (4 × 250 mm; Dionex) at the Biopolymer Research Center for Advanced Materials at Sejong University. The samples were diluted with distilled water, filtered through a 0.45-μm polyvinylidene fluoride (PVDF) syringe filter, and 20 μL volumes were injected for analysis. Elution was performed using a 100 mM NaOH solution at a flow rate of 1.0 mL/min in an isocratic mode.

### 2.9 In Vitro Digestion of Theanderose

To assess the *in vitro* digestibility of theanderose, a digestion test was conducted following the AOAC 2009.01 method.^23^ The digestion conditions utilized Megazyme’s porcine pancreatic α-amylase and amyloglucosidase to hydrolyze carbohydrates. Theanderose, along with four other carbon sources (corn starch, maltotriose, erythrose, and raffinose), was incubated in maleate buffer (pH 6.0) at 37 °C for 16 h. The degree of degradation of each carbon source was determined by quantifying the released glucose using HPAEC-PAD.

### 2.10 Effects of Theanderose on the Growth of *Bifidobacterium* and *Lactobacillus*

To assess the effects of theanderose on the growth of *Bifidobacterium* and *Lactobacillus* strains, a specific growth medium was prepared for each bacterial strain. De Man, Rogosa, and Sharpe (MRS) (Difco, Becton, Franklin Lakes) and *B. lactis* (BL) (Difco, Becton, Franklin Lakes) media were used for culturing *Lactobacillus* spp. and *Bifidobacterium* spp., respectively. Bacterial strains were precultured without shaking at 37 °C for 18 h in 10 mL of the respective medium. The culture medium was a modified basal medium (mBM) comprising yeast extract (10 g/L), peptone (10 g/L), NaCl (0.66 g/L), K_2_HPO_4_ (0.264 g/L), MgSO_4_·7H_2_O (0.066 g/L), hemin solution (0.033 mL/L), Vitamin K1 (66 μL/L), cysteine-HCl (0.33 g/L), and sodium thioglycolate (0.5 g/L). The medium was autoclaved at 121 °C for 15 min. Carbon sources (such as theanderose, erlose, turanose, trehalulose, raffinose, and maltotriose) were added at a concentration of 0.5% (w/v) to each 96-well plate, following filtration through a 0.2 μm membrane. Glucose was used as a control for growth, and media without any carbon source served as a negative control for each bacterial test. The cultures were incubated in an anaerobic atmosphere using the Gas Pack system (GasPak EZ, BD). Cell cultures were collected after 24 h to assess the growth patterns of the strains. Growth patterns were determined by measuring the optical density (OD) at 600 nm using a spectrophotometer (DU 730; Beckman Coulter Inc., Brea, United States). Each analysis was conducted in duplicate.

### 2.11 Gut Microbiome Effects of Theanderose on Fecal Fermentation

This study was approved by the Sejong University Institutional Review Board (SJU-HR-E-2017–009). Participation was voluntary and included written informed consent. Koreans who had not been prescribed antibiotics for one month before the experiment and who were free of chronic diseases, such as diabetes or hypertension, were recruited. Fresh stool samples from 20 healthy donors were immediately transferred into anaerobic workstation and were prepared within an hour. To minimize matrix effects, the samples were thoroughly homogenized and anaerobic conditions were maintained throughout the preparation. Two types of samples were prepared: one served as a control (nontreatment), and the other was treated with glucose, maltose, sucrose, and theanderose. As theanderose is a glucosylated form of sucrose, sucrose was selected as the basic comparative sample to better assess the impact of glucosylation on gut microbiota. Fecal fermentation of each sample was conducted according to the method outlined by Li et al.^24^ The gut microbiome was analyzed using a barcoded high-throughput sequencing approach described by Earth Microbiome Project^25^ (EMP). Total genomic DNA was extracted from each fecal sample using the DNeasy PowerSoil kit (Qiagen, Hilden, Germany), following the manufacturer’s protocol. The extracted DNA was stored at −80 °C. The V4 region of the 16S rRNA gene was amplified from the genomic DNA using 515 forward and 806 reverse primers with barcodes. The PCR amplicon products were purified using the NucleoSpin PCR cleanup kit (Macherey-Nagel, Duren, Germany) and subsequently sequenced on an Illumina MiSeq platform (2 × 300 bp paired-end reads). Paired-end FASTQ files were processed using quantitative insights into microbial ecology 2 (QIIME 2) (version amplicon-2024.2; https://qiime2.org). Demultiplexed reads were processed using the divisive amplicon denoising algorithm 2 (DADA2) packages for quality filtering and denoising, resulting in the generation of amplicon sequence variants (ASVs). Taxonomy assignment of each ASV was performed using the SILVA database v. 132. Beta-diversity was assessed using UniFrac distances and principal coordinate analysis (PCoA), and taxonomy annotation was performed using QIIME to visualize the microbial communities between the control and theanderose-treated groups. Baseline fecal samples were clustered based on the relative abundance at the genus levels using the Jensen-Shannon divergence (JSD) distance and the Partitioning Around Medoids (PAM) clustering algorithm in the R environment.^26^ Calinski-Harabasz (CH) index was assessed to determine the optimal number of clusters.^27^ Permutational multivariate analysis of variance (PERMANOVA) was used to determine significant differences in bacterial structures, utilizing distance metrics to confirm the strength and statistical significance of sample groupings in the PCoA plots.^28^ We carried out a linear discriminant analysis effect size (LEfSe) analysis to detect significant differences in bacterial taxonomies, with the α value for the factorial Kruskal–Wallis test set at 0.05 and a logarithmic LDA score threshold of 3.0.^29^

### 2.12 Effects of Theanderose on Metabolites in Fecal Fermentation Using U-HPLC MS/MS

The fecal sample (200 μL) was extracted by mixing an equal volume of 40% of acetonitrile, followed by vigorous shaking for 1 min, then incubated at −20 °C for 2 h. The resulting mixture was centrifuged at 4 °C and 13,000 × *g* for 15 min, followed by homogenization using a Tissue Lyser (Qiagen) at 30 Hz for 10 min. The supernatant was subjected to LC-MS/MS to profile the metabolites, with quality control (QC) samples used in parallel. The metabolites derived from fecal microbiota were analyzed using a U-HPLC system (Vanquish U-HPLC, Thermo Fisher Scientific, Waltham, MA, USA), coupled to a quadrupole mass spectrometer (Thermo Scientific Orbitrap Exploris 120 high-resolution/accurate mass spectrometer, interfaced with a heated electrospray ionization (H-ESI) source), at the Biopolymer Research Center for Advanced Materials (BRCAM, Sejong University, Seoul, Republic of Korea). The procedures are performed as described previously.^30^ For the chromatographic separation, the prepared samples (10 μL) were injected into a C18 column (Waters, ACQUITY UPLC BEH C18, 2.1 × 100 mm, 1.7 μm, Waters Corp., Milford, MA, USA), with the column temperature maintained at 45 °C throughout the acquisition period. The mobile phase was eluted by a gradient of water (A) and acetonitrile (B), containing 0.1% acetic acid, with a gradient dilution profile of 98% A (0–2 min), 98–2% A (2–15 min), 2% A (15–17 min), 2–98% A (17–18 min), and 98% A (18–20 min), at a flow rate of 0.3 mL/min. The mass spectrum conditions included a heated capillary of 325 °C, a vaporizer temperature of 350 °C, a spray voltage of 3.5 kV in positive mode, and 2.5 kV in negative mode; the sheath gas, aux gas, and sweep gas were set at 50, 10, and 1 psi, respectively. The HCD collision energy was set at 15, 30, and 60%, and the scan range covered 55–800 *m*/*z* in full scan, with a resolution of 120,000 for MS1 and 15,000 for MS2. The raw MS data were processed using Xcalibur version 4.6 and Compound Discoverer 3.3 (Thermo Fisher Scientific, Waltham, MA, USA).

### 2.13 Statistical Analysis

Data are presented as means with corresponding standard deviations, derived from triplicate independent experiments. Statistical evaluations were performed using SPSS software (version 12.0 K for Windows; SPSS Inc., Chicago, IL, USA).

## 3 Results and Discussion

### 3.1 Determination of Product Distribution in the *Dd*AS Reactant

**Figure 1:**
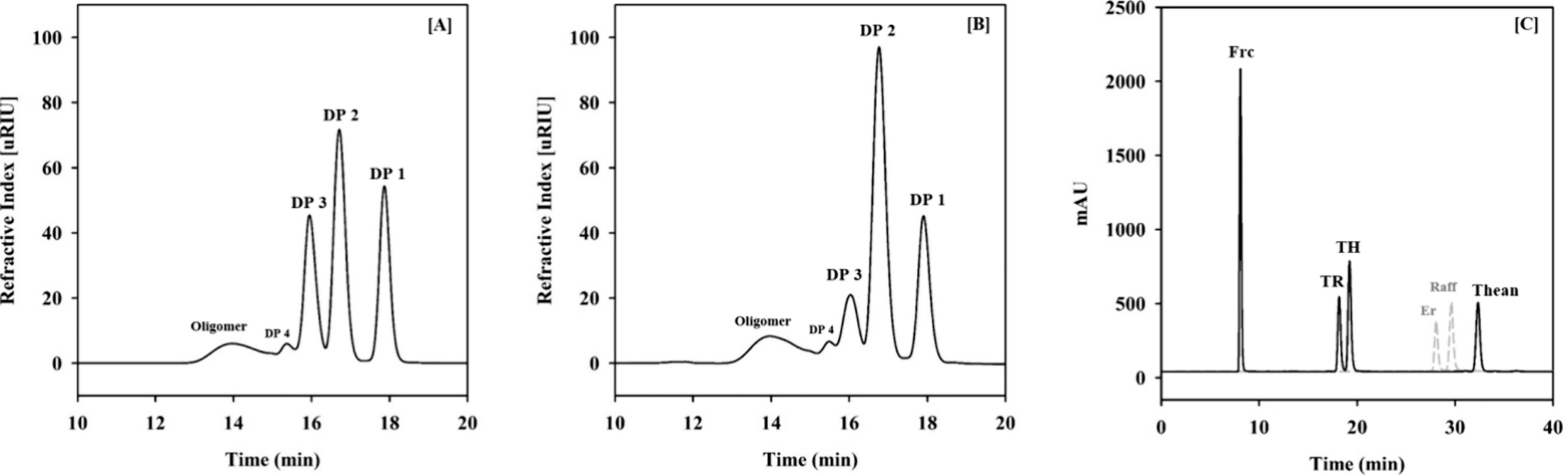
Distribution of constituent sugars in the products is assessed following a reaction in 50 mM sodium phosphate buffer (pH 7.5) at 35 °C for 120 h. For this constituent sugar distribution study, the reaction is conducted using 400 U/L *D. deserti* (*Dd*AS) with 2.0 M sucrose as the substrate. (A) High-performance size exclusion chromatography-refractive index (HPSEC-RI) chromatogram of *Dd*AS reaction products; (B) HPSEC-RI chromatogram of *Dg*AS reaction products; and (C) high-performance liquid chromatography-evaporative light scattering detection (HPLC-ELSD) chromatogram of *Dd*AS reaction products. Frc, fructose; TR, turanose; TH, trehalulose; Er, erlose; Raff, raffinose; Thean, theanderose. Due to the characteristics of HPLC-ELSD, different types of sugars exhibit varying levels of sensitivity. The gray dashed lines in (C) indicate the expected retention times for Er (erlose) and Raff (raffinose), which are not detected in the *Dd*AS reaction products.

*Dd*AS significantly reduced the amount of soluble and insoluble α-glucan produced by ASase and generated lower molecular weight glucoside products (primarily sucrose isomers such as turanose and trehalulose), when 400 U/L *Dd*AS was reacted with a 2.0 M sucrose solution in 50 mM sodium phosphate buffer (pH 7.5) for 120 h at 35 °C^15^. Analysis of reaction products across various sucrose concentrations revealed that 2.0 M was optimal for maximizing the production of desired oligosaccharides while minimizing the formation of byproducts such as glucose, fructose, and insoluble glucans (Figure S2). The *Dd*AS reactant was analyzed using HPSEC-RI and HPLC-ELSD to efficiently analyze the small-molecule glucoside products (Figure 1). The HPSEC-RI results demonstrated that ASase reacted with sucrose to produce monosaccharides (DP 1), disaccharides (DP 2), trisaccharides (DP 3), tetrasaccharides (DP 4), and oligosaccharides. *Dd*AS had a lower production rate for DP 2 compared to *Dg*AS, whereas DP 3 had a significantly higher production rate than that of *Dg*AS (Figure 1A and B). The HPLC-ELSD analysis of the *Dd*AS reaction revealed the peaks of fructose, trehalulose, and turanose that are produced by ASases, and an unknown peak that is not produced by *Dg*AS (Figure 1C). HPLC-ELSD analysis demonstrated that *Dd*AS converts 100.6 and 154.7 g/L of truanose and trehalulose from sucrose as the sole substrate, respectively, which is consistent with the previous studies.^15^ However, the retention time of the unknown peak did not align with that of HPLC-ELSD with DP 3, such as erlose and raffinose. *Np*AS variants with modifications in loops 3, 4, and 7 effectively produced erlose by stably binding its fructofuranosyl portion with structural alterations in the +2 subsite. Additionally, they produced panose by altering the arrangement of glucopyranosyl units in the subsite +1/+2 and modifying the hydrogen-bonding network.^18^ Moreover, the *Np*AS mutations G396S and T398V sterically hindered binding at the +2/+3 subsites of oligosaccharides longer than DP3, consistent with the results of the *Dd*AS reaction. *Dd*AS replaced Gly396 and Thr398 in loop 7 of *Np*AS with Gly391 and Ala393, respectively, and replaced *Np*AS Cys445 with Arg44. These mutations were located near the +2 subsite, causing an alteration in the fluidity of loop 7 in the B’ domain. This resulted in variations in the reaction and production properties of ASases.^18^ Therefore, it could be speculated that the variations in subsite residues of *Dd*AS might result in different production properties compared to other ASases. Although ASases preferentially converted glucose to a glucosyl acceptor molecule with an α-1,4 linkage, they could also convert it to an α-1,6 linkage based on the conformation of the acceptor and reaction conditions.^17^ Therefore, we speculated that the unknown trisaccharide was a putative theanderose with an α-1,6 linkage of glucose to sucrose.

### 3.2 Effects of Temperature and PH on Enzymatic Production Yields of Putative Theanderose

**Figure 2:**
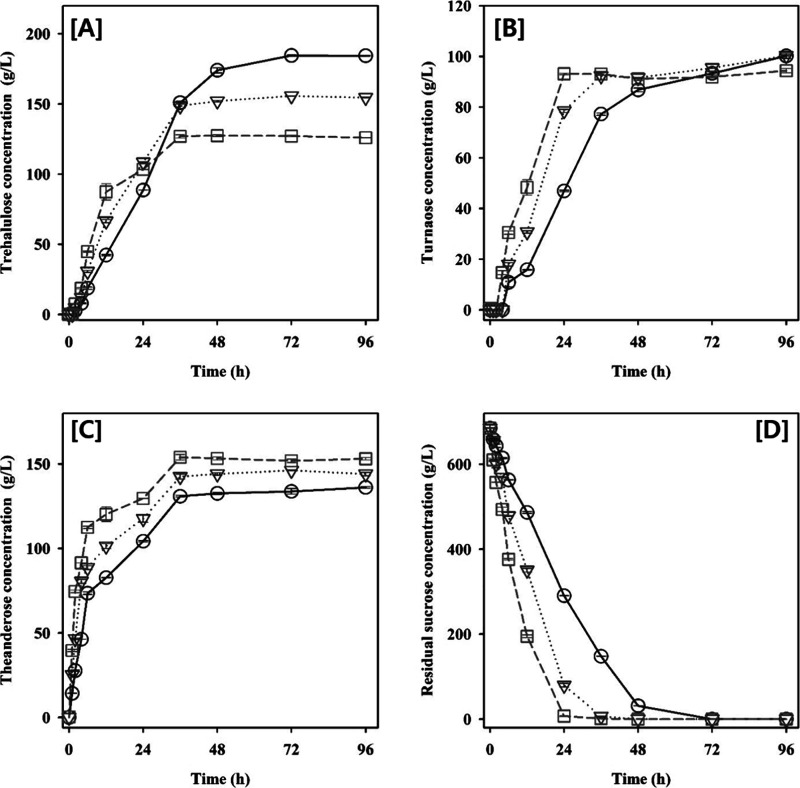
Time course of product formation and substrate consumption by DdAS at different temperatures. The reactions were performed under varying temperature conditions (circles, 30 °C; inverted triangles, 35 °C; and squares, 40 °C) for 96 h in 50 mM sodium phosphate buffer (pH 7.5) using 2.0 M sucrose as a substrate. (A) Trehalulose production; (B) turanose production; (C) theanderose production; (D) residual sucrose concentration. The measurements were made in triplicate, and values are expressed as the mean ± SEM.

**Figure 3:**
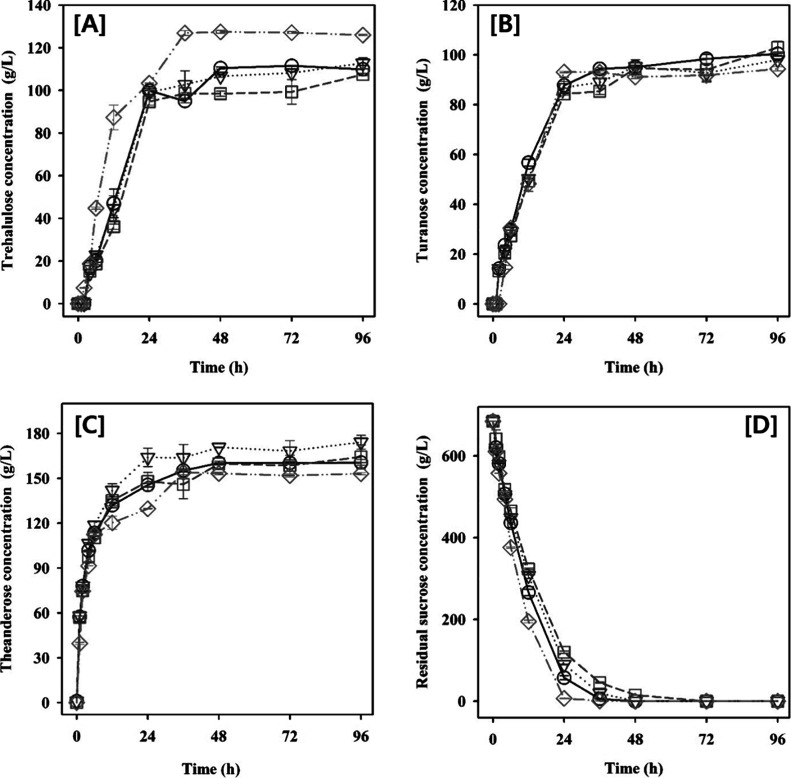
Time course of product formation and substrate consumption by DdAS under different pH conditions. The reactions were performed under varying pH conditions (circles, 50 mM sodium acetate pH 6.0; inverted triangles, 50 mM sodium phosphate pH 6.0; diamonds, 50 mM sodium phosphate pH 7.5; and squares, 50 mM glycine-NaOH pH 9.0) for 96 h at 40 °C using 2.0 M sucrose as a substrate. (A) Trehalulose production; (B) turanose production; (C) theanderose production; (D) residual sucrose concentration. The measurements were made in triplicate, and values are expressed as the mean ± SEM.

*Dd*AS was reacted with 2.0 M sucrose as the sole substrate at various temperatures and pH levels to assess the effects of reaction temperature and pH on putative theanderose production (Figures 2 and 3). There were no significant variations in the pattern of sucrose consumption and production rates of turanose, trehalulose, and putative theanderose based on reaction pH when *Dd*AS was reacted with 2.0 M sucrose for 96 h at 40 °C in various buffers (Figure 3). Previous studies have shown that enzyme activity remains above 80% efficiency in sodium acetate buffer (pH 6.0), sodium phosphate buffer (pH 7.5), and glycine-NaOH buffer (pH 9.0).^15,19^ Based on these findings, we selected these pH ranges for our experiments. Additionally, sodium phosphate buffer (pH 6.0) was included to provide a comparison with the sodium acetate buffer (pH 6.0). Furthermore, we previously assessed the thermostability of *Dd*AS and found that its half-life at 40 °C was 62 h, but this decreased rapidly to 6 h at 45 °C. This information was considered when determining the temperature range for theanderose synthesis. Although the optimum reaction temperature of *Dd*AS was 35 °C,^15^ it consumed all the sucrose within approximately 24 h at 40 °C when *Dd*AS was reacted with 2.0 M sucrose in 50 mM sodium phosphate buffer at pH 7.5 for 96 h at 30, 35, and 40 °C. ASases exhibit varying ratios of hydrolysis, transglycosylation, and isomerization reactions primarily in response to temperature changes, while pH differences generally affect these reaction proportions to a lesser extent.^31^ These results indicated that the ability of *Dd*AS to consume sucrose was more sensitive to reaction temperature than to reaction pH. The optimal bioconversion yield of the putative theanderose was 174.1 g/L when *Dd*AS was reacted with 2.0 M sucrose in 50 mM sodium phosphate buffer (pH 6) at 40 °C. In general, the optimal reaction temperature and pH for ASase were determined by measuring the amount of reducing sugars produced in the initial reaction using the DNS method,^32^ which were often inconsistent with the optimal conditions for longer reactions, such as transglycosylation and isomerization.^33^ The production efficiency of turanose was not significantly affected by the reaction temperature; however, the production efficiency of trehalulose reduced significantly with increasing reaction temperature (Table 1). The biosynthesis of theanderose using α-glucosidase was reported, but it was characterized by low productivity^34^ (Table 2). In contrast, levansucrase exhibited significantly higher productivity in the biosynthesis of theanderose.^9^ However, despite its high productivity, levansucrase also used isomaltose as an acceptor, and as the reaction time increased, the concentration of theanderose decreased, affecting the efficiency of the reaction process. In contrast, DdAS not only exhibited higher productivity than α-glucosidase but also did not require isomaltose or additional acceptor molecules. Moreover, the amount of theanderose remained stable during the reaction, facilitating more consistent process control compared to levansucrase (Table 2).

**Table 1:** Effects of pH and Temperature on the Production of Theanderose, Trehalulose, and Turanose By *Dd*AS after a 96 h Reaction

reaction-affecting factors	theanderose production (g/L)	trehalulose production (g/L)	turanose production (g/L)
**pH**			
sodium acetate 6.0	160.6 ± 2.0	109.9 ± 2.0	99.1 ± 0.8
sodium phosphate 6.0	174.1 ± 4.7	112.8 ± 2.5	98.0 ± 0.4
sodium phosphate 7.5	153.1 ± 0.7	125.9 ± 0.3	94.4 ± 0.8
glycine-NaOH 9.0	164.3 ± 8.1	107.3 ± 0.5	103.0 ± 2.5
**temperature (°C)**			
30	136.1 ± 0.5	184.3 ± 0.2	100.2 ± 1.0
35	144.2 ± 1.0	154.5 ± 0.3	100.3 ± 0.3
40	153.1 ± 0.7	125.9 ± 0.3	94.4 ± 0.8

**Table 2:** Comparison of Enzymes and Reaction Conditions for Theanderose Synthesis

					**substrate (M)**				
**enzyme**	**origin**	**reaction temperature (°C)**	**reaction pH**	**reaction time (h)**	**sucrose**	**isomaltose**	**residual sucrose (M)**	**theanderose productivity (g/L·h)**	**references**
amylosucrase	*Deinococcus deserti*	40	6.0	48	2.00		0	3.6	this study
levansucrase	*Bacillus subtilis* CECT39	37	6.0	3	0.61	0.59	0.26	42.1	(9)
α-glucosidase	*Metschnikowia reukaufii*			72	1.46			0.078	(54)
α-glucosidase	*Bacillus subtilis* SAM1606	60	6.0	48	1.75			1.3	(34)

**Figure 4:**
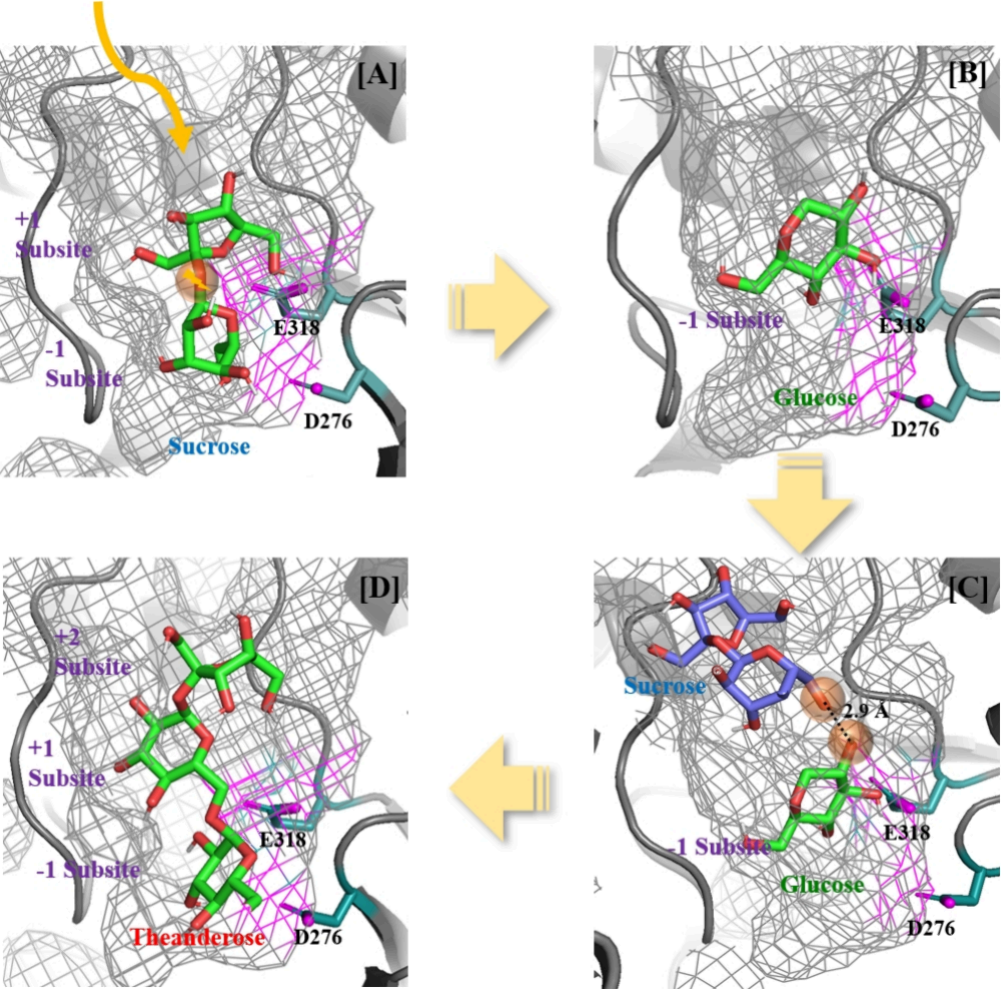
Predicted the three-dimensional (3D) structure of proteins and protein–ligand binding using the AlphaFold2 and AutoDock Vina programs, respectively. (A) Initial sucrose binding, (B) glucose intermediate formation, (C) sucrose acceptor binding, and (D) novel linkage formation.

Additionally, the production of putative theanderose increased, while that of trehalulose decreased. *Dg*AS had a very weak electron density of fructosyl attachments at the acceptor binding site, resulting in a higher amount of trehalulose compared to *Np*AS by accepting various fructose tautomers.^35^ Moreover, the percent concentration of fructose tautomers in aqueous solutions was linearly related to the temperature.^36^ Therefore, it could be that the yield of trehalulose production in *Dd*AS was temperature-dependent because the concentration of fructose tautomers suitable for trehalulose binding was altered by the reaction temperature. Interestingly, the sucrose consumption pattern of *Dd*AS and the production pattern of putative theanderose were similar. However, the production of putative theanderose did not increase as the amount of trehalulose decreased. ASase could transfer glucose from sucrose to the hydroxyl group (−OH) present in glucosyl compounds and −OH of various compounds.^12^ This indicated that *Dd*AS might transfer glucose from sucrose as a donor molecule to sucrose, with −OH groups as acceptor molecules. Therefore, the putative biosynthetic process of theanderose was simulated through molecular docking (Figure 4). Initially, the sucrose entered the active site of *Dd*AS, where the glucosyl and fructosyl motifs of sucrose were located at the −1 and +1 subsites, respectively (Figure 4A). Fructose at the +1 subsite was released by D276 and E318 (the active sites of *Dd*AS) and glucose at the −1 subsite covalently bound to E318 to form the *Dd*AS-glucose complex (Figure 4B). Molecular docking predicted the conformation of a ligand and assessed its binding affinity, resulting in various ligand conformations (poses).^37^ Molecular docking simulations demonstrated that sucrose (as an acceptor molecule) formed various poses, with the majority of them located away from the catalytic site. The active site of ASase comprises +1 to +5 subsites, to which the glucosyl motif of sucrose might have bound.^18^ Among the various poses in the docking simulation, we observed that the glucosyl O6’ position of sucrose was closest to the *Dd*AS-glucose complex at 2.9 Å, a distance at which the reaction could have occurred (Figure 4C). Finally, the O6’ glucosyl motif of sucrose at the +1 subsite bound to O1 of the glucosyl motif of the *Dd*AS-glucose complex to form 6^G^-α-d-glucosyl-sucrose (Figure 4D). Therefore, it is assumed that *Dd*AS could biosynthesize theanderose with 1,6-bonded glucose using sucrose as the acceptor and donor molecule through molecular docking and altering the reactants based on the reaction conditions (Figure S3). To further validate the docking results, Molecular Dynamics (MD) simulations were conducted with theanderose in the binding site (Figure S4). The results of the RMSF analysis demonstrated that the presence of theanderose led to a noticeable reduction in flexibility at key regions, particularly near Arg441, which is associated with the substrate binding sites (Figure S4A). The reduction in RMSF values suggested that the binding of theanderose conferred additional stability to these regions, reducing their dynamic fluctuations. As shown in Figure S8B, theanderose formed a key hydrogen bond with Arg441 at a distance of 2.6 Å, which was critical for the stabilization of these subsites (Figure S4B).

### 3.3 Purification and Structural Identification of Putative Theanderose in the *Dd*AS Reactant

The sample for purification was subjected to a 24-h reaction with 2.0 M sucrose using 0.3 mg/mL *Dd*AS in a 50 mM sodium phosphate buffer (pH 6.0) at 40 °C. These conditions were optimal for producing putative theanderose. The resulting putative theanderose was purified to >98% purity using MPLC (Figure S5). The purified putative theanderose was analyzed using electrospray ionization mass spectrometry (ESI-MS), revealing a molecular ion peak at *m*/*z* 503.30 [M-H]^−^, which corresponded to a trisaccharide (C_18_H_32_O_16_) (Figure S6). The NMR spectra (^1^H NMR and ^13^C NMR) of the putative theanderose were listed in Table 3. Two-dimensional (2D) NMR analysis was used to confirm the bonding structure of the putative theanderose. The δ_H_ and δ_C_ data of the putative theanderose were assessed using 1D NMR and HSQC analyses that identified the positions of directly bonded carbon and hydrogen (Figure S7). A more detailed 2D NMR analysis using HMBC was performed to associate the chemical shifts of carbon and hydrogen separated by two or three bonds.^38^ This confirmed the linkage structure of putative theanderose by determining the linkage between the carbon in putative theanderose and hydrogen in glucose and fructose. HMBC analysis confirmed 12 cross sections in the putative theanderose structure, specifically at C-2″/H-1′, C-2″/H-1″, C-3′/H-1′, C-5/H-1, C-6’/H-1, C-1″/H-3″, C-6″/H-4″, C-4/H-6, C-3″/H-1″, C-2’/H-4′, C-6’/H-4′, and C-6/H-4. In addition, the ^1^H–^1^H COSY analysis confirmed the position of the correlating hydrogen spectrum, which demonstrated nine cross sections of putative theanderose at H-1/H-2, H-2/H-1, H-1’/H-2′, H-4’/H-5′/H-2’/H-1′, H-4″/H-3″, H-4″/H-5″, H-3″/H-4″, and H-3″/H-4″ (Figure S8). HMBC correlation analysis revealed the α-d-Glc-1(1→6′)-α-d-Glc-2 linkage as an isomeric proton correlation of H-1 (δ_H_ 4.88, *J* = 3.70 Hz) for the Glc-1 moiety and C-6′ (δ_C_ 65.6) for the Glc-2 moiety. The α-d-Glc-(1′→2″)-β-d-Fru linkage was supported by the HMBC correlation of the fructose residue to H-1′ (δ_H_ 5.35, *J* = 3.82 Hz), whereas the C-2″ of Glc-2 (δ_C_ 103.8) was supported by the HMBC correlation. Therefore, the trisaccharide purified from the *Dd*AS reaction was identified as theanderose, which is an α-d-glucopyranosyl-(1→6)-α-d-glucopyranosyl-(1→2)-β-d-fructofuranoside.

**Table 3:**
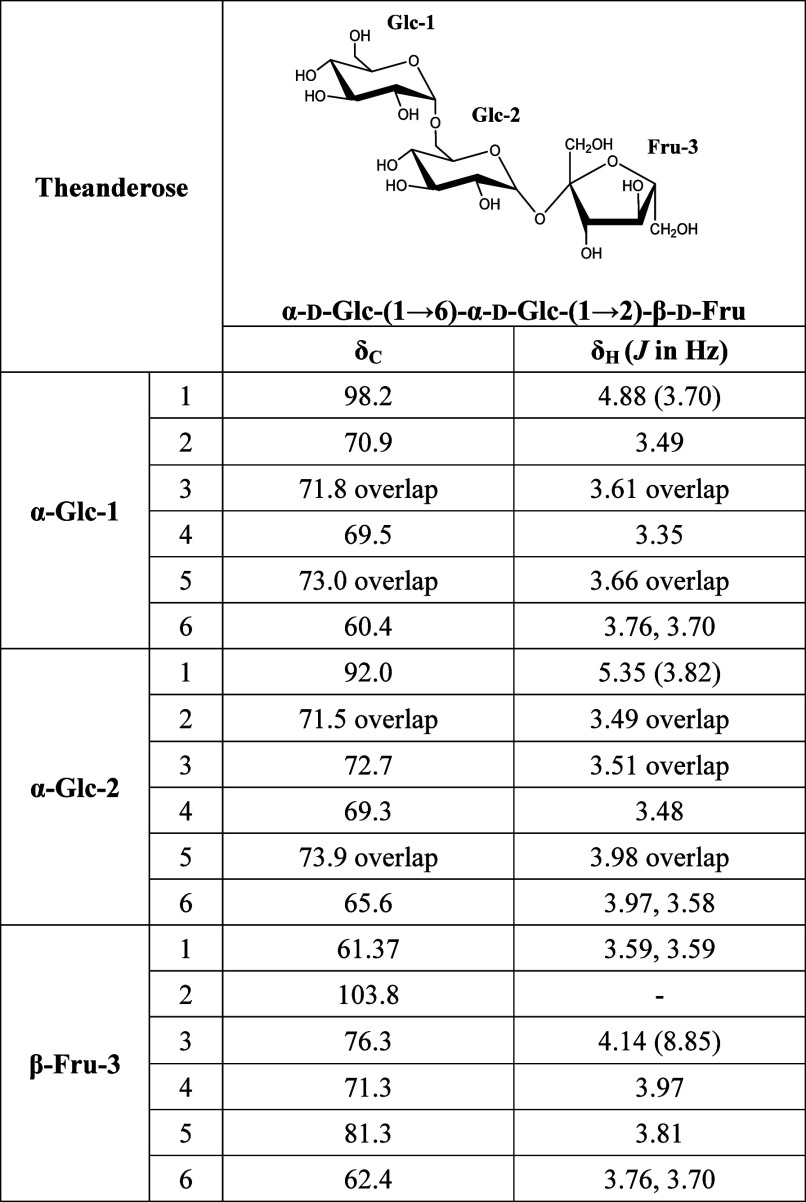
^1^H-NMR and ^13^C-NMR Chemical Shifts (δ_C_, ppm) and Coupling Constants (δ_H_, *J* in Hz) Determined Using One- and Two-Dimensional (1D) and (2D) Nuclear Magnetic Resonance (NMR) Spectroscopy of Trisaccharide in D_2_O

### 3.4 *In Vitro* Digestibility of Theanderose and Its Potential as a Carbon Source for Probiotic Strains

**Figure 5:**
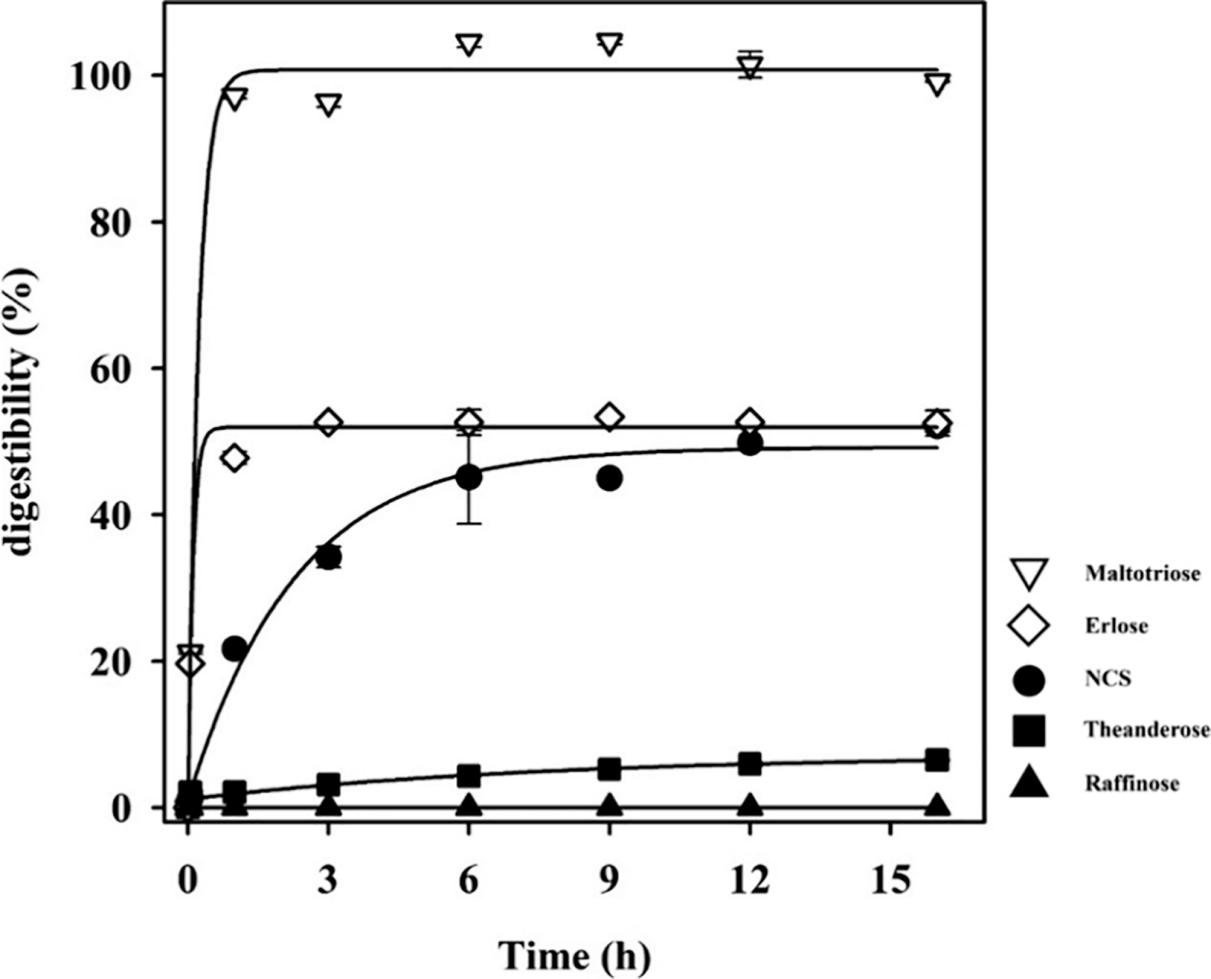
Digestibility (%) of theanderose and four other carbohydrates is assessed over 16 h at 37 °C in an *in vitro* digestion model using α-amylase and α-glucosidase.

Carbohydrate-based prebiotics should not be broken down into simple sugars by digestive enzymes for use by the gut microbiota.^39^ The *in vitro* digestibility of theanderose was compared with that of erlose (α-d-glucopyranosyl-(1→4)-α-d-glucopyranosyl-(1→2)-β-d-fructofuranoside) and raffinose, which were structurally similar to theanderose (Figure 5). Erlose was rapidly degraded into glucose and sucrose using porcine pancreatic α-amylase and amyloglucosidase from *Aspergillus niger*, whereas theanderose and raffinose were not degraded for a long time. Amyloglucosidase primarily degraded erlose by hydrolyzing it from the nonreducing end, as both enzymes hydrolyzed α-1,4-d-glucosidic linkages in endo- and exotypes, respectively, and were unable to hydrolyze sucrose.^40^ Although amyloglucosidase could hydrolyze the terminal α-1,4 and α-1,6 d-glucose residues,^40^ it could barely hydrolyze theanderose. A previous study exhibited that theanderose was not completely degraded in human saliva and porcine pancreas, whereas it was 3.7% degraded to fructose and isomaltose in an artificial gastric juice environment.^10^ Additionally, theanderose was also degraded to fructose, glucose, and sucrose by approximately 50.2% using rat intestinal acetone powder (RIAP). The result indicated that theanderose was partially degraded by α-glucosidase, sucrase, and β-galactosidase in RIAP.^41^ Therefore, theanderose was not completely degraded by host digestive enzymes, making it a prebiotic material that could be used by gut microbes.

**Figure 6:**
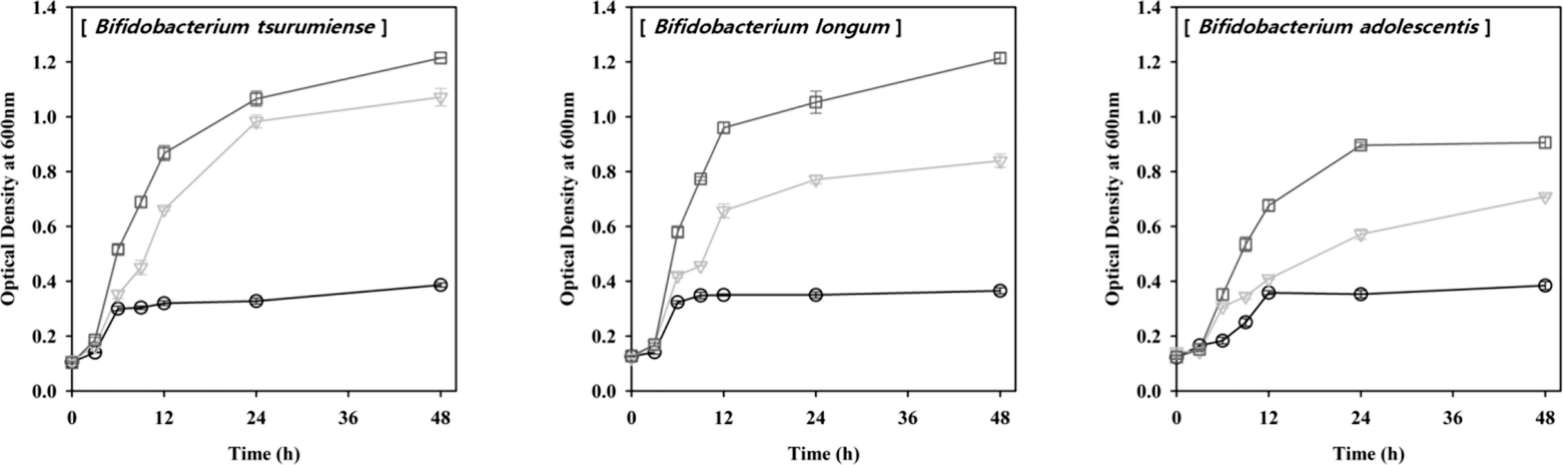
Growth curves of three *Bifidobacterium* species using different carbon sources. Cultures were incubated at 37 °C for 48 h. Growth was measured by optical density at 600 nm. Carbon sources: squares, theanderose; inverted triangles, glucose; and circles, blank (no added carbon source).

The potential prebiotic effect of theanderose was determined by measuring the growth rate of probiotic strains, such as *Lactobacillus* and *Bifidobacterium* on various carbon sources (Table S1). Although the probiotic strain used glucose as a carbon source, the growth rate varied depending on the type of carbon source. All *Bifidobacterium* strains used theanderose as a carbon source, which facilitated their growth, whereas *L. rhamnosus* GG and *Weissella cibaria* barely used theanderose as a carbon source. We examined the growth curves of three *Bifidobacterium* species: *B. tsurumiense*, *B. longum*, and *B. adolescentis* (Figure 6). All three species demonstrated significantly higher growth rates when utilizing theanderose compared to glucose. *B. tsurumiense* and *B. longum* showed particularly robust growth on theanderose for 48 h compared to growth on glucose. *B. adolescentis* also exhibited enhanced growth on theanderose compared to glucose, although the difference was less pronounced than in the other two species. A previous study displayed that theanderose facilitated the growth of *B. adolescentis*, *B. breve*, *B. infantis*, and *B. longum*, while it did not affect the growth of *B. bifidum*.^10^*Bifidobacterium* had various carbohydrate-active enzymes that facilitated the degradation of nondigestible carbohydrate prebiotic materials.^42^*Bifidobacterium* used theanderose, erlose, raffinose, and maltotriose for growth, and β-fructofuranosidase, α-glucosidase, oligo-1,6-glucosidase, and α-galactosidase for degradation.^43^*B. bifidum* differed from other *Bifidobacterium* strains in its ability to degrade host-derived glucans, but lacked certain carbohydrate-active enzymes, making it less fermentable for other carbohydrates.^44^ Therefore, it was speculated that theanderose enhanced the gut environment by facilitating the growth of *Bifidobacterium* in the gut, without being degraded by digestive enzymes.

### 3.5 Evaluating the Potential Prebiotic Effects of Theanderose through Analysis of Fecal Microbiota Composition

**Figure 7:**
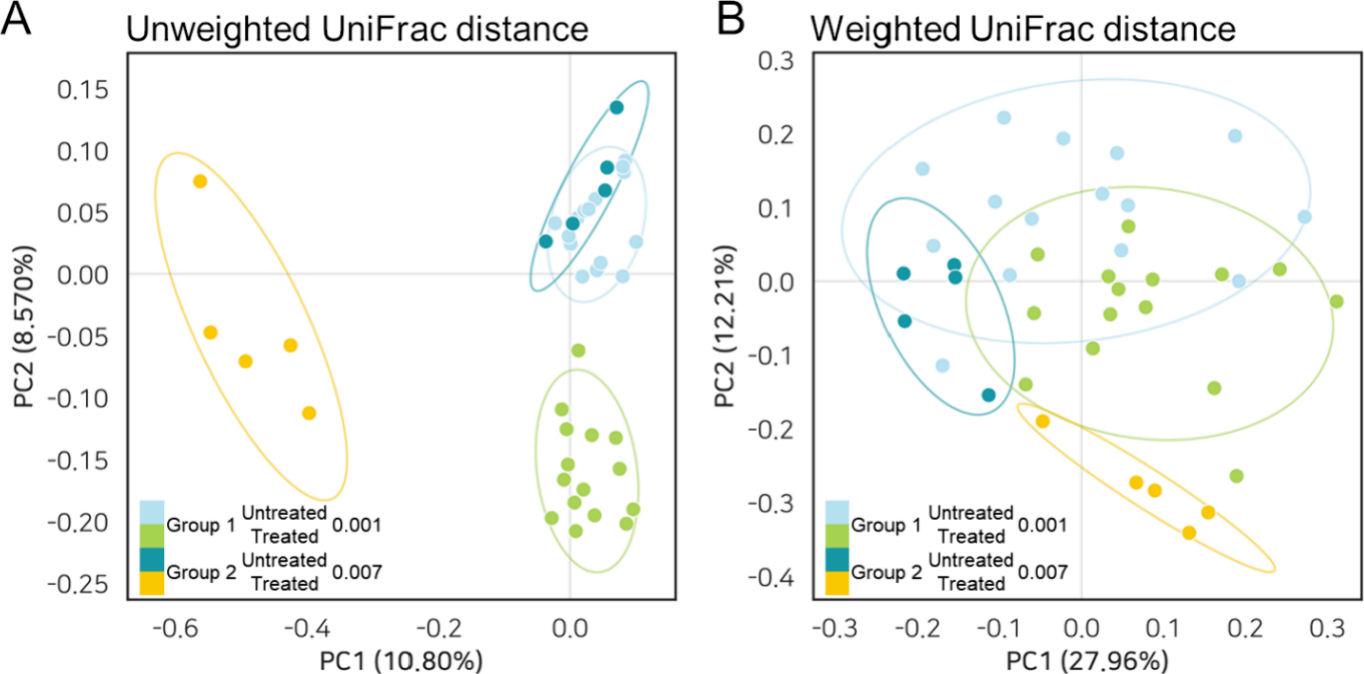
Beta diversity analysis and principal coordinate analysis (PCoA) plots based on (A) Unweighted (B) and weighted UniFrac distance demonstrating the diversity among fecal samples. PERMANOVA was used to test dissimilarity. There are no significant differences based on sex; however, alterations in groups based on the presence or absence of the Prevotella family in untreated fecal samples are significant.

**Figure 8:**
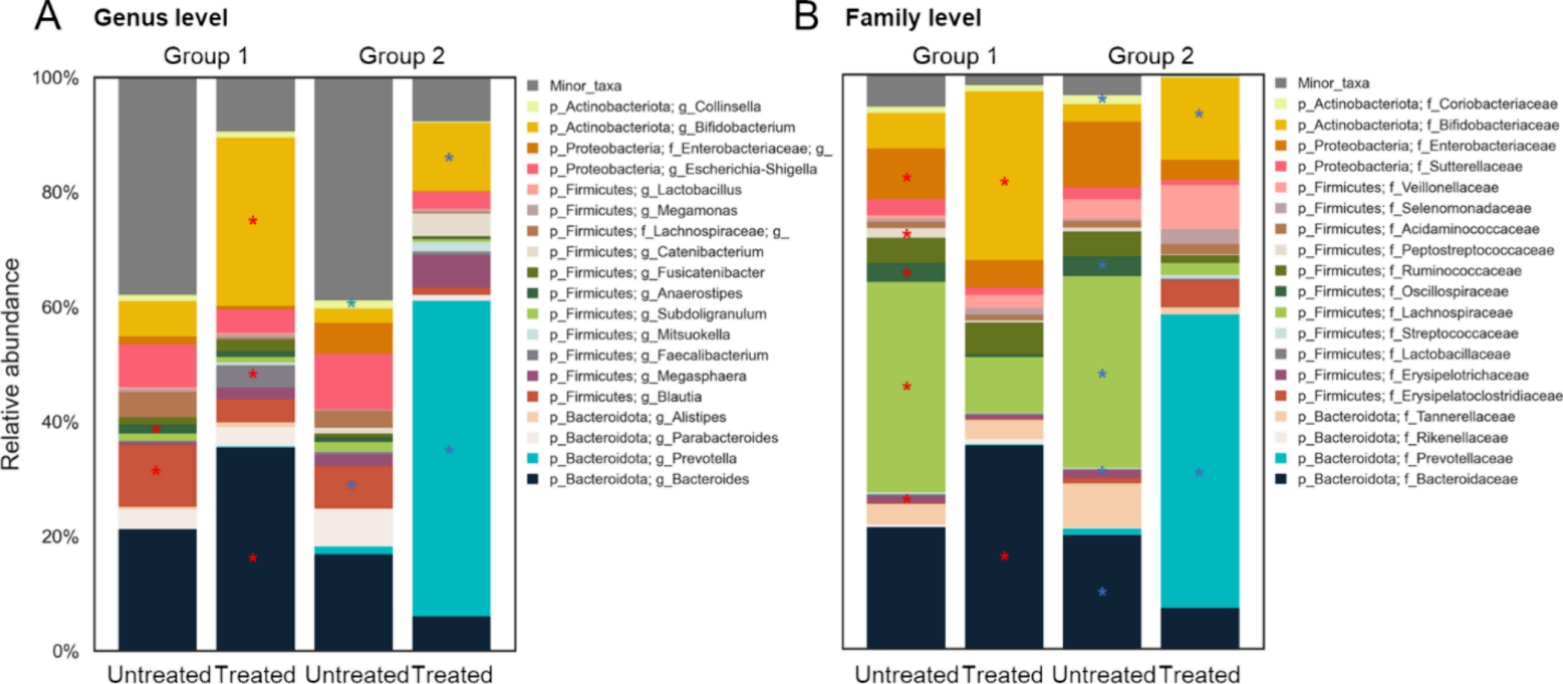
Responses of the gut microbiota composition to theanderose interventions at the (A) genus and (B) family level. Asterisks indicate differentially enriched taxa following the theandrose intervention (red: Group 1, blue: Group 2). The LDA scores indicate the effect sizes of the taxonomy with difference relative abundances theanderose intervention in (A) Group 1 and (B) Group 2 (using LDA > 3.0).

Fecal fermentation is a method of cofermenting feces from various donors and prebiotic materials in an *in vitro* anaerobic culture system to determine alterations in the gut microbiome and analyze the effects of prebiotics on the gut microbiome.^45^ Fecal fermentation was performed by adding theanderose to the feces of 20 individuals (both male and female) of varying ages to assess the theanderose-induced alterations in gut microbiota. The fecal fermentations supplemented with glucose, maltose, sucrose, and theanderose showed significant changes in gut microbiota compared to the control. In the fermentation supplemented with sugars including theanderose, both *Bifidobacerium* and *Faecalibacterium* were significantly increased (LDA score >3.0). However, the gut microbiota changes in the theanderose-treated fermentation, such as the increase in *Prevotella*, were different from those observed with other sugars (Figure S11). Additionally, while glucose, maltose, and sucrose could all serve as excellent carbon sources for microbes, they were absorbed in the gastrointestinal tract and used in energy metabolism before they could be utilized by gut microbiota.^2^ Therefore, it was hypothesized that theanderose, which was not broken down by digestive enzymes, was broken down into glucose and sucrose by the gut microbiota and led to changes in the composition of the gut microbiota. To reduce individual variability and better understand the complexity of the gut microbiota, we determined the enterotype of each subject. Using the Jensen–Shannon divergence (JSD) distance and the partitioning around medoids (PAM) clustering algorithm, we clustered the samples based on their relative abundance at the genus level. The optimal number of clusters was estimated using the Calinski–Harabasz (CH) index (Figure S10A). This analysis resulted in the separation of the microbiota structures into two distinct clusters (Figure S10B), with 15 subjects classified as *Bacteroides*-dominant (group 1) and 5 subjects as *Prevotella*-dominant (group 2). It is important to note that enterotyping is primarily a method to simplify the complexity of the gut microbiota, rather than to define rigidly distinct clusters. The diversity among theanderose-treated fecal samples was expressed through beta diversity analysis and PCoA plot. The gut microbiota communities of groups 1 (PERMANOVA, Unweighted; *p* < 0.001, Weighted; *p* < 0.001) and group 2 (PERMANOVA, Unweighted; *p* < 0.007, Weighted; *p* < 0.007) were structurally separated following the intervention of theanderos (Figure 7A, B). Theanderose-induced alterations in the microbial community were compared at the genus level, with a distinction between enterotype (Figure 8). Interestingly, *Prevotellaceae* was rare in group 1 of the untreated samples compared to group 2, whereas *Bifidobacteriaceae* in group 1 and *Prevotellaceae* in group 2 were significantly increased by theanderose. Additionally, theanderose increased the proportion of *Bifidobacteriaceae* in group 2. *Bifidobacteriaceae* comprised various anaerobic and facultative anaerobic bacteria that include the genus *Bifidobacterium* in the gut microbiota, whose growth was facilitated by the use of theanderose.^10^*Bifidobacterium* strains had numerous enzymes that could degrade prebiotics, thereby facilitating their selective increase in response to prebiotic intake.^43^ Raffinose (galactosyl sucrose), which was similar in structure to theanderose (glucosyl sucrose), enhanced the composition of the gut microbiome by facilitating the proliferation of the beneficial bacteria such as *Bifidobacterium* and *Lactobacillus*.^5^ However, diets rich in raffinose reduced the relative abundance of *Prevotellaceae*.^46^*Prevotellaceae* was associated with plant dietary fiber metabolism, and specifically, the *Prevotella* genus facilitated the production of SCFAs, such as propionate.^47^ While 16S rRNA gene-based sequencing is a standard tool for analyzing gut microbiota composition and structure, it does have limitations in differentiating between viable and nonviable microorganisms, which can potentially lead to an inaccurate representation of the active microbial community. To address this, we conducted additional metabolome analysis using U-HPLC MS/MS to support our findings on the differences in microbiome structure between groups. We found that theanderose tends to increase the production of propionate and lactate specifically in the *Prevotella*-dominant enterotype, as compared to the *Bacteroides*-dominant enterotype (Figure S10). SCFAs positively influence the gut environment, leading to improved health outcomes.^48^ A high relative abundance of *Prevotella* was associated with enhanced blood glucose levels in mice and cardiovascular disease risk factor profiles in humans.^49^ Theanderose increased the relative abundance of *Prevotellaceae* and *Bifidobacteriaceae* compared to raffinose, and demonstrated that *Prevotellaceae* effectively used theanderose than that of *Bifidobacteriaceae*. This might be because of the unique glycosyl structure of theanderose. Theanderose significantly reduced the proportion of *Lachnospiraceae* and *Ruminococcaceae,* regardless of the group (Figure 8). The *Lachnospiraceae* exhibited both beneficial and adverse effects on host health owing to the species/strain-level diversity and metabolite variations among its members.^50^ Increases in the genera *Blautia* and *Mediterraneibacter gnavus* in the *Lachnospiraceae* family were observed in patients with inflammatory bowel disease and primary sclerosing cholangitis.^50^ Increases in *Lachnospiraceae* were also associated with metabolic diseases, such as obesity and type 2 diabetes.^51^*Lachnospiraceae* might contribute to obesity by increasing energy absorption by producing SCFAs. Additionally, the metabolites of *Lachnospiraceae* might contribute to insulin resistance, lipid metabolism abnormalities, and more.^51^ Gut bacteria in *Ruminococcaceae* were closely associated with obesity,^52^ and the abundance of *Ruminococcaceae* bacteria was increased in obese and type 2 diabetes model mice and in mice fed a high-fat diet (HFD).^52^ The HFD mice treated with enzymatically recrystallized chestnut starch had reduced adipocytes and reduced abundance of *Ruminococcaceae* bacteria compared with that of the untreated HFD mice.^53^ Although there were uncertainties in predicting the effects of alterations in certain gut microbiomes on host health, theanderose increased the abundance of communities that positively affected host health and decreased the abundance of communities that negatively affected host health. Future *in vivo* experiments in animal models are required for the physiological assessment of these findings.

This study demonstrated that *Dd*AS can efficiently biosynthesize novel trisaccharides compared to other ASases, based on its enzymatic properties. A flowchart detailing the experimental workflow was included in the Supporting Information (Figure S12). The novel trisaccharide biosynthesized by *Dd*AS was identified as theanderose with an α-d-glucopyranosyl-(1→6)-α-d-glucopyranosyl-(1→2)-β-d-fructofuranose through NMR analysis. Theanderose exhibited resistance to degradation *in vitro* using α-amylase and amyloglucosidase, and was selectively used as a carbon source by the probiotic strains of *Bifidobacterium* spp. Moreover, fecal fermentation analysis demonstrated that theanderose significantly increased the relative abundances of *Prevotellaceae* and *Bifidobacteriace* in the gut microbiota. Therefore, this study demonstrated the efficient biosynthesis of theanderose into *Dd*AS, a potential prebiotic that could modulate the gut microbiota. Consequently, our findings indicate a promising avenue for the mass production of theanderose and its application as a novel contender in the food, cosmetic, and pharmaceutical industries.

## Supporting Information Available

Figure S1, SDS-PAGE analysis of the *Dd*AS protein purified by Ni-NTA affinity chromatography; Figure S2, Composition of reaction products at various sucrose concentrations; Figure S3, Mechanism of theanderose synthesis by amylosucrase; Figure S4, Molecular dynamics simulation results and binding site visualization of the *Dd*AS complex with theanderose; Figure S5, Targeted separation of DdAS reaction product samples used for MPLC; Figure S6, HPLC-MS chromatograms of purified theanderose; Figure S7, HSQC of theanderose dissolved in D_2_O; Figure S8, HMBC and COSY of theanderose dissolved in D_2_O; Table S1, Growth of *Lactobacillus* & *Bifidobacterium* was verified using diverse carbon sources, with cultures incubated at 37 °C over a 24 h period; Figure S9, Changes in the gut microbiota composition at the genus and family level in response to treatments with glucose, maltose, sucrose, and theanderose; Figure S10, Gut enterotype classification of subjects; Figure S11, Comparison of amount of SCFAs between the control and theanderose-treated groups for *Bacteroides* and *Prevotella* types; Figure S12, Flowchart of amylosucrase production, theanderose synthesis, and prebiotic effect evaluation (PDF)

## Abbreviations used

*Dd*AS: ASase from *Deinococcus deserti*
*Dg*AS: ASase from *Deinococcus geothermalis*
*Np*AS: ASase from *Neisseria polysaccharea*
HPAEC-PAD: high-performance anion-exchange chromatography-pulsed amperometric detection
HPLC-ELSD: high-performance liquid chromatography-evaporative light scattering detection
MPLC: medium-pressure liquid chromatography
NMR: nuclear magnetic resonance
QIIME 2: quantitative insights into microbial ecology 2
DADA2: divisive amplicon denoising algorithm 2
ASVs: amplicon sequence variants
PCoA: principal coordinate analysis

## References

[ref1] Liu X.; Ke L.; Lei K.; Yu Q.; Zhang W.; Li C.; Tian Z. Antibiotic-induced gut microbiota dysbiosis has a functional impact on purine metabolism. BMC Microbiol 2023, 23 (1), 187 10.1186/s12866-023-02932-8. 37442943 PMC10339580

[ref2] Gibson G. R.; Hutkins R.; Sanders M. E.; Prescott S. L.; Reimer R. A.; Salminen S. J.; Scott K.; Stanton C.; Swanson K. S.; Cani P. D.; et al. Expert consensus document: The International Scientific Association for Probiotics and Prebiotics (ISAPP) consensus statement on the definition and scope of prebiotics. Nat. Rev. Gastroenterol Hepatol 2017, 14 (8), 491–502. 10.1038/nrgastro.2017.75. 28611480

[ref3] Davani-Davari D.; Negahdaripour M.; Karimzadeh I.; Seifan M.; Mohkam M.; Masoumi S.J.; Berenjian A.; Ghasemi Y. Prebiotics: Definition, types, sources, mechanisms, and clinical applications. Foods 2019, 8 (3), 92 10.3390/foods8030092. 30857316 PMC6463098

[ref4] Amorim C.; Silvério S. C.; Cardoso B. B.; Alves J. I.; Pereira M. A.; Rodrigues L. R. *In vitro* fermentation of raffinose to unravel its potential as prebiotic ingredient. Lwt 2020, 126, 109322 10.1016/j.lwt.2020.109322.

[ref5] Xiao M.; Ren X.; Cheng J.; Fu X.; Li R.; Zhu C.; Kong Q.; Mou H. Structural characterization of a novel fucosylated trisaccharide prepared from bacterial exopolysaccharides and evaluation of its prebiotic activity. Food Chem. 2023, 420, 136144 10.1016/j.foodchem.2023.136144. 37060669

[ref6] Makelainen H.; Hasselwander O.; Rautonen N.; Ouwehand A. C. Panose, a new prebiotic candidate. Lett. Appl. Microbiol 2009, 49 (6), 666–672. 10.1111/j.1472-765X.2009.02698.x. 19874483

[ref7] Elango D.; Rajendran K.; Van der Laan L.; Sebastiar S.; Raigne J.; Thaiparambil N. A.; El Haddad N.; Raja B.; Wang W.; Ferela A.; Chiteri K. O.; Thudi M.; Varshney R. K.; Chopra S.; Singh A.; Singh A. K.; et al. Raffinose family oligosaccharides: Friend or foe for human and plant health?. Front Plant Sci. 2022, 13, 829 10.3389/fpls.2022.829118. PMC8891438 35251100

[ref8] Nagao M.; Oku K.; Minami A.; Mizuno K.; Sakurai M.; Arakawa K.; Fujikawa S.; Takezawa D. Accumulation of theanderose in association with development of freezing tolerance in the moss *Physcomitrella patens*. Phytochemistry 2006, 67 (7), 702–709. 10.1016/j.phytochem.2006.01.031. 16527318

[ref9] Ruiz-Aceituno L.; Sanz M. L.; de Las Rivas B.; Munoz R.; Kolida S.; Jimeno M. L.; Moreno F. J. Enzymatic synthesis and structural characterization of theanderose through transfructosylation reaction catalyzed by levansucrase from *Bacillus subtilis* CECT 39. J. Agric. Food Chem. 2017, 65 (48), 10505–10513. 10.1021/acs.jafc.7b03092. 29131629

[ref10] Shimokawa H.; Takeda Y.; Wada K.; Shimizu T. In vitro digestion and utilization of theanderose by various intestinal bacteria. J. Jpn. Soc. Nutr Food Sci. 1993, 46 (1), 69–76. 10.4327/jsnfs.46.69.

[ref11] Potocki de Montalk G.; Remaud-Simeon M.; Willemot R. M.; Sarcabal P.; Planchot V.; Monsan P. Amylosucrase from *Neisseria polysaccharea*: novel catalytic properties. FEBS Lett. 2000, 471 (2–3), 219–223. 10.1016/S0014-5793(00)01406-X. 10767427

[ref12] Wu J. Y.; Ding H. Y.; Luo S. Y.; Wang T. Y.; Tsai Y. L.; Chang T. S. Novel glycosylation by amylosucrase to produce glycoside anomers. Biology 2022, 11 (6), 822 10.3390/biology11060822. 35741343 PMC9220500

[ref13] Tian Y.; Xu W.; Zhang W.; Zhang T.; Guang C.; Mu W. Amylosucrase as a transglucosylation tool: From molecular features to bioengineering applications. Biotechnol Adv. 2018, 36 (5), 1540–1552. 10.1016/j.biotechadv.2018.06.010. 29935268

[ref14] Seo D. H.; Jung J. H.; Jung D. H.; Park S.; Yoo S. H.; Kim Y. R.; Park C. S. An unusual chimeric amylosucrase generated by domain-swapping mutagenesis. Enzyme Microb Technol. 2016, 86, 7–16. 10.1016/j.enzmictec.2016.01.004. 26992787

[ref15] Bae J.; Jun S. J.; Chang P. S.; Yoo S. H. A unique biochemical reaction pathway towards trehalulose synthesis by an amylosucrase isolated from *Deinococcus deserti*. N Biotechnol 2022, 70, 1–8. 10.1016/j.nbt.2022.03.004. 35339700

[ref16] Rha C. S.; Kim H. G.; Baek N. I.; Kim D. O.; Park C. S. Using amylosucrase for the controlled synthesis of novel isoquercitrin glycosides with different glycosidic linkages. J. Agric. Food Chem. 2020, 68 (47), 13798–13805. 10.1021/acs.jafc.0c05625. 33175543

[ref17] Jung Y. S.; Kim H. G.; Oh S. M.; Lee D. Y.; Park C. S.; Kim D. O.; Baek N. I. Synthesis of alpha-linked glucosides from soybean isoflavone aglycones using amylosucrase from *Deinococcus geothermalis*. J. Agric. Food Chem. 2023, 71 (5), 2430–2437. 10.1021/acs.jafc.2c07778. 36701419

[ref18] Verges A.; Cambon E.; Barbe S.; Moulis C.; Remaud-Simeon M.; Andre I. Novel product specificity toward erlose and panose exhibited by multisite engineered mutants of amylosucrase. Protein Sci. 2017, 26 (3), 566–577. 10.1002/pro.3106. 28019698 PMC5326559

[ref19] Im J.-K.; Seo D.-H.; Yu J. S.; Yoo S.-H. Efficient and novel biosynthesis of myricetin α-triglucoside with improved solubility using amylosucrase from *Deinococcus deserti*. Int. J. Biol. Macromol. 2024, 273, 133205 10.1016/j.ijbiomac.2024.133205. 38885871

[ref20] Jumper J.; Evans R.; Pritzel A.; Green T.; Figurnov M.; Ronneberger O.; Tunyasuvunakool K.; Bates R.; Žídek A.; Potapenko A.; Bridgland A.; Meyer C.; Kohl S. A. A.; Ballard A. J.; Cowie A.; Romera-Paredes B.; Nikolov S.; Jain R.; Adler J.; Back T.; Petersen S.; Reiman D.; Clancy E.; Zielinski M.; Steinegger M.; Pacholska M.; Berghammer T.; Bodenstein S.; Silver D.; Vinyals O.; Senior A. W.; Kavukcuoglu K.; Kohli P.; Hassabis D. Highly accurate protein structure prediction with AlphaFold. Nature 2021, 596 (7873), 583–589. 10.1038/s41586-021-03819-2. 34265844 PMC8371605

[ref21] Eberhardt J.; Santos-Martins D.; Tillack A. F.; Forli S. AutoDock Vina 1.2. 0: New docking methods, expanded force field, and python bindings. J. Chem. Inf Model 2021, 61 (8), 3891–3898. 10.1021/acs.jcim.1c00203. 34278794 PMC10683950

[ref22] Páll S.; Zhmurov A.; Bauer P.; Abraham M.; Lundborg M.; Gray A.; Hess B.; Lindahl E. Heterogeneous parallelization and acceleration of molecular dynamics simulations in GROMACS. J. Chem. Phys. 2020, 153 (13), 134110 10.1063/5.0018516. 33032406

[ref23] McCleary B. V.; Sloane N.; Draga A.; Lazewska I. Measurement of total dietary fiber using AOAC Method 2009.01 (AACC International Approved Method 32–45.01): evaluation and updates. Cereal Chem. 2013, 90 (4), 396–414. 10.1094/CCHEM-10-12-0135-FI.

[ref24] Li L.; Abou-Samra E.; Ning Z.; Zhang X.; Mayne J.; Wang J.; Cheng K.; Walker K.; Stintzi A.; Figeys D. An *in vitro* model maintaining taxon-specific functional activities of the gut microbiome. Nat. Commun. 2019, 10 (1), 4146 10.1038/s41467-019-12087-8. 31515476 PMC6742639

[ref25] Thompson L. R.; Sanders J. G.; McDonald D.; Amir A.; Ladau J.; Locey K. J.; Prill R. J.; Tripathi A.; Gibbons S. M.; Ackermann G.; et al. A communal catalogue reveals Earth’s multiscale microbial diversity. Nature 2017, 551 (7681), 457–463. 10.1038/nature24621. 29088705 PMC6192678

[ref26] Arumugam M.; Raes J.; Pelletier E.; Le Paslier D.; Yamada T.; Mende D. R.; Fernandes G. R.; Tap J.; Bruls T.; Batto J.-M.; et al. Enterotypes of the human gut microbiome. Nature 2011, 473 (7346), 174–180. 10.1038/nature09944. 21508958 PMC3728647

[ref27] Caliński T.; Harabasz J. A dendrite method for cluster analysis. Commun. Stat 1974, 3 (1), 1–27. 10.1080/03610927408827101.

[ref28] Anderson M. J. A new method for non-parametric multivariate analysis of variance. Austral Ecology 2001, 26 (1), 32–46. 10.1111/j.1442-9993.2001.01070.pp.x.

[ref29] Segata N.; Izard J.; Waldron L.; Gevers D.; Miropolsky L.; Garrett W. S.; Huttenhower C. Metagenomic biomarker discovery and explanation. Genome Biol. 2011, 12 (6), R60 10.1186/gb-2011-12-6-r60. 21702898 PMC3218848

[ref30] Park G.; Kim S.; Lee W.; Kim G.; Shin H. Deciphering the impact of defecation frequency on gut microbiome composition and diversity. Int. J. Mol. Sci. 2024, 25 (9), 4657 10.3390/ijms25094657. 38731876 PMC11083994

[ref31] Seo D.-H.; Yoo S.-H.; Choi S.-J.; Kim Y.-R.; Park C.-S. Versatile biotechnological applications of amylosucrase, a novel glucosyltransferase. Food Sci. Biotechnol 2020, 29 (1), 1–16. 10.1007/s10068-019-00686-6. 31976122 PMC6949346

[ref32] Emond S.; Potocki-Veronese G.; Mondon P.; Bouayadi K.; Kharrat H.; Monsan P.; Remaud-Simeon M. Optimized and automated protocols for high-throughput screening of amylosucrase libraries. J. Biomol Screen 2007, 12 (5), 715–723. 10.1177/1087057107301978. 17517906

[ref33] Seo D.-H.; Jung J.-H.; Park C.-S. Fluorescence detection of the transglycosylation activity of amylosucrase. Anal. Biochem. 2017, 532, 19–25. 10.1016/j.ab.2017.05.028. 28577993

[ref34] Okada M.; Nakayama T.; Noguchi A.; Yano M.; Hemmi H.; Nishino T.; Ueda T. Site-specific mutagenesis at positions 272 and 273 of the *Bacillus* sp. SAM1606 α-glucosidase to screen mutants with altered specificity for oligosaccharide production by transglucosylation. J. Mol. Catal. B Enzym 2002, 16 (5–6), 265–274. 10.1016/S1381-1177(01)00071-6.

[ref35] Guerin F.; Barbe S.; Pizzut-Serin S.; Potocki-Veronese G.; Guieysse D.; Guillet V.; Monsan P.; Mourey L.; Remaud-Simeon M.; Andre I.; et al. Structural investigation of the thermostability and product specificity of amylosucrase from the bacterium *Deinococcus geothermalis*. J. Biol. Chem. 2012, 287 (9), 6642–6654. 10.1074/jbc.M111.322917. 22210773 PMC3307298

[ref36] Barclay T.; Ginic-Markovic M.; Johnston M. R.; Cooper P.; Petrovsky N. Observation of the *keto* tautomer of D-fructose in D_2_O using ^1^H NMR spectroscopy. Carbohydr. Res. 2012, 347 (1), 136–141. 10.1016/j.carres.2011.11.003. 22129837 PMC3254704

[ref37] Meng X. Y.; Zhang H. X.; Mezei M.; Cui M. Molecular docking: a powerful approach for structure-based drug discovery. Curr. Comput. Aided Drug Des 2011, 7 (2), 146–157. 10.2174/157340911795677602. 21534921 PMC3151162

[ref38] Jadeja Y.; Kapadiya K.; Shah A.; Khunt R. Importance of HMBC and NOE 2D NMR techniques for the confirmation of regioselectivity. Magn. Reson. Chem. 2016, 54 (1), 75–80. 10.1002/mrc.4315. 26307589

[ref39] Anggraeni A.; Mini-Review A. The potential of raffinose as a prebiotic. IOP Conf Ser. Earth Environ. Sci. 2022, 980, 012033 10.1088/1755-1315/980/1/012033.

[ref40] Zhang B.; Dhital S.; Gidley M. J. Synergistic and antagonistic effects of α-amylase and amyloglucosidase on starch digestion. Biomacromolecules 2013, 14 (6), 1945–1954. 10.1021/bm400332a. 23647443

[ref41] Seo J. M.; Lamothe L. M.; Shin H.; Austin S.; Yoo S. H.; Lee B. H. Determination of glucose generation rate from various types of glycemic carbohydrates by mammalian glucosidases anchored in the small intestinal tissue. Int. J. Biol. Macromol. 2020, 154, 751–757. 10.1016/j.ijbiomac.2020.03.154. 32194128

[ref42] Wang H.; Huang X.; Tan H.; Chen X.; Chen C.; Nie S. Interaction between dietary fiber and *Bifidobacteria* in promoting intestinal health. Food Chem. 2022, 393, 133407 10.1016/j.foodchem.2022.133407. 35696956

[ref43] Petrova P.; Petrov K. Chapter 7 - Prebiotic–Probiotic Relationship: The Genetic Fundamentals of Polysaccharides Conversion by *Bifidobacterium*and *Lactobacillus*Genera. In Food Bioconversion, Grumezescu A. M., Holban A. M., Eds.; Academic Press, 2017; pp 237–278.

[ref44] Katoh T.; Ojima M. N.; Sakanaka M.; Ashida H.; Gotoh A.; Katayama T. Enzymatic adaptation of *Bifidobacterium bifidum* to host glycans, viewed from glycoside hydrolyases and carbohydrate-binding modules. Microorganisms 2020, 8 (4), 481 10.3390/microorganisms8040481. 32231096 PMC7232152

[ref45] Lee D. H.; Seong H.; Chang D.; Gupta V. K.; Kim J.; Cheon S.; Kim G.; Sung J.; Han N. S. Evaluating the prebiotic effect of oligosaccharides on gut microbiome wellness using in vitro fecal fermentation. NPJ. Sci. Food 2023, 7 (1), 18 10.1038/s41538-023-00195-1. 37160919 PMC10170090

[ref46] Chen J.; Li Z.; Wang X.; Fan B.; Deng F.; D.Yu H.; Ze X.; Zhu L.; Yin Y.; Chen Y.; Zhao J.; Yang Y.; Wang X.; Jensen P. A.; et al. Isomaltooligosaccharides sustain the growth of *Prevotella* both *in vitro* and in animal models. Microbiol Spectr 2022, 10 (6), e0262121 10.1128/spectrum.02621-21. 36377936 PMC9769830

[ref47] Chung W. S. F.; Walker A. W.; Bosscher D.; Garcia-Campayo V.; Wagner J.; Parkhill J.; Duncan S. H.; Flint H. J. Relative abundance of the *Prevotella* genus within the human gut microbiota of elderly volunteers determines the inter-individual responses to dietary supplementation with wheat bran arabinoxylan-oligosaccharides. BMC Microbiol 2020, 20 (1), 283 10.1186/s12866-020-01968-4. 32928123 PMC7490872

[ref48] Portincasa P.; Bonfrate L.; Vacca M.; De Angelis M.; Farella I.; Lanza E.; Khalil M.; Wang D. Q.; Sperandio M.; Di Ciaula A. Gut microbiota and short chain fatty acids: Implications in glucose homeostasis. Int. J. Mol. Sci. 2022, 23 (3), 1105 10.3390/ijms23031105. 35163038 PMC8835596

[ref49] Gálvez E. J. C.; Iljazovic A.; Amend L.; Lesker T. R.; Renault T.; Thiemann S.; Hao L.; Roy U.; Gronow A.; Charpentier E.; Strowig T.; et al. Distinct polysaccharide utilization determines interspecies competition between intestinal *Prevotella* spp. Cell Host Microbe 2020, 28 (6), 838–852 e836. 10.1016/j.chom.2020.09.012. 33113351

[ref50] Abdugheni R.; Wang W. Z.; Wang Y. J.; Du M. X.; Liu F. L.; Zhou N.; Jiang C. Y.; Wang C. Y.; Wu L.; Ma J.; et al. Metabolite profiling of human-originated *Lachnospiraceae* at the strain level. iMeta 2022, 1 (4), e58 10.1002/imt2.58. 38867908 PMC10989990

[ref51] Vacca M.; Celano G.; Calabrese F. M.; Portincasa P.; Gobbetti M.; De Angelis M. The controversial role of human gut *Lachnospiraceae*. Microorganisms 2020, 8 (4), 573 10.3390/microorganisms8040573. 32326636 PMC7232163

[ref52] Lakshmanan A. P.; Al Zaidan S.; Bangarusamy D. K.; Al-Shamari S.; Elhag W.; Terranegra A. Increased relative abundance of *Ruminoccocus* is associated with reduced cardiovascular risk in an obese population. Front Nutr 2022, 9, 849005 10.3389/fnut.2022.849005. 35571941 PMC9097523

[ref53] Feng J.; Ma H.; Huang Y.; Li J.; Li W. *Ruminococcaceae*_UCG-013 promotes obesity resistance in mice. Biomedicines 2022, 10 (12), 3272 10.3390/biomedicines10123272. 36552029 PMC9776008

[ref54] Garcia-Gonzalez M.; Plou F. J.; Cervantes F. V.; Remacha M.; Poveda A.; Jiménez-Barbero J.; Fernandez-Lobato M. Efficient production of isomelezitose by a glucosyltransferase activity in *Metschnikowia reukaufii* cell extracts. Microbial Biotechnology 2019, 12 (6), 1274–1285. 10.1111/1751-7915.13490. 31576667 PMC6801145

