# Supplementary material for: Efficient Biosynthesis of Theanderose, a Potent Prebiotic, Using Amylosucrase from Deinococcus deserti
Source: J Agric Food Chem. 2024 Oct 31;72(45):25197–209. doi: 10.1021/acs.jafc.4c05763 (PMC11565756; doi:10.1021/acs.jafc.4c05763)
Supplement: Supplementary file 1 — jf4c05763_si_001.pdf [file jf4c05763_si_001.pdf]

Supplementary materials

Efficient biosynthesis of theanderose, a potent prebiotic, using amylosucrase from *Deinococcus deserti*

Jeon-Uk Kang[a], Yun-Sang So[a], Gyungcheon Kim[a], WonJune Lee[a], Dong-Ho Seo[a], Hak-Dong Shin[a,]*, Sang-Ho Yoo[a,]*

[a]Department of Food Science & Biotechnology, and Carbohydrate Bioproduct Research Center, Sejong University, Seoul 05006, Republic of Korea

* Corresponding author:

Hakdong Shin, Tel.: +82-2-6935-2525. Fax: +82-2-3408-4319. E-mail: hshin@sejong.ac.kr.

Sang-Ho Yoo, Tel.: +82 2 3408 3221; fax: +82 2 3408 4319; e-mail: shyoo@sejong.ac.kr.

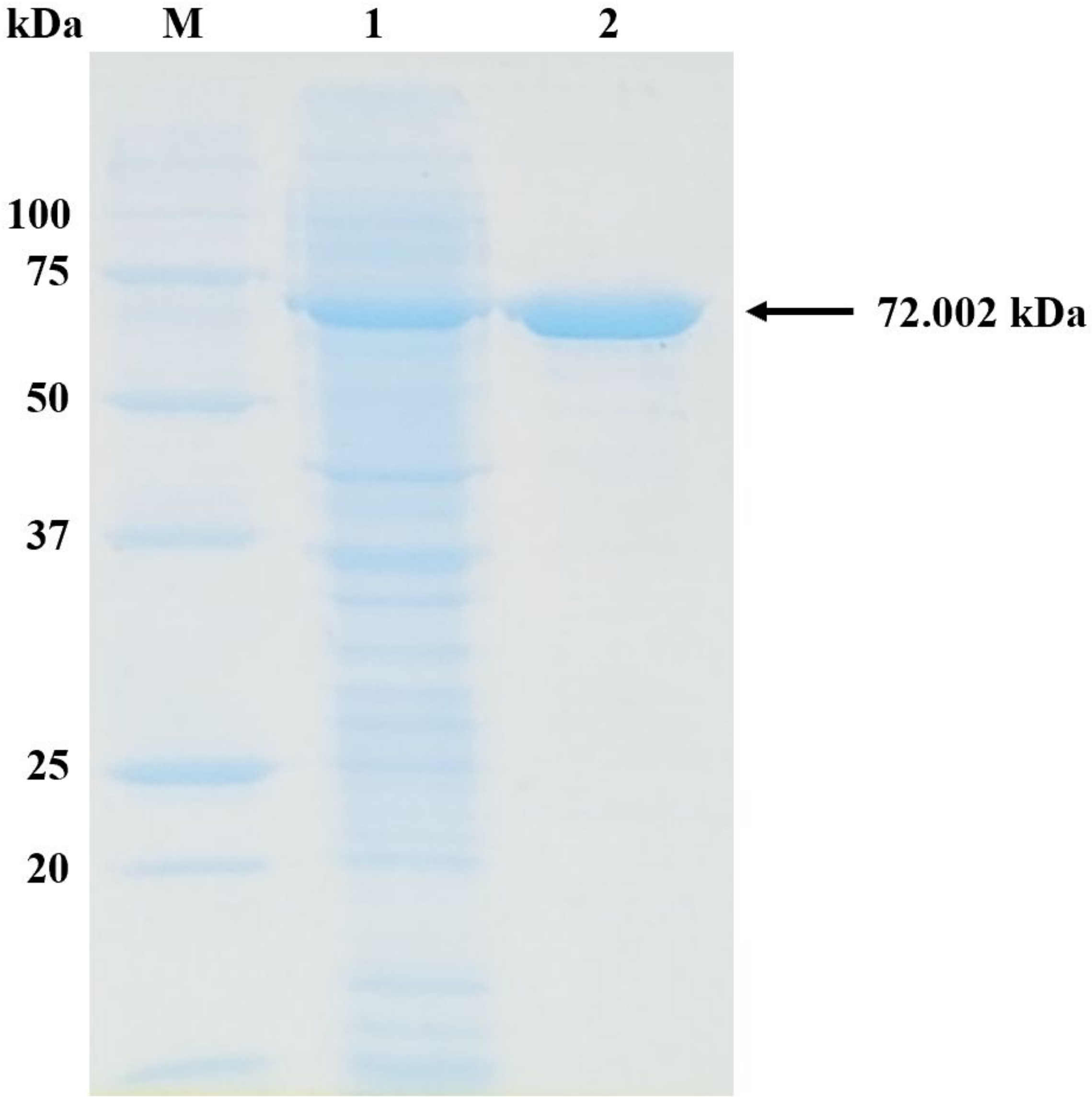

Figure S1. SDS-PAGE analysis of the *Dd*AS protein purified by Ni-NTA affinity chromatography. Lane M: Protein size marker; Lane 1: Cell extract of *E. coli* BL21(DE3) harboring *Dd*AS expression plasmid; Lane 2: Purified *Dd*AS after Ni-NTA affinity chromatography. The purified *Dd*AS protein shows a single band at approximately 72 kDa.

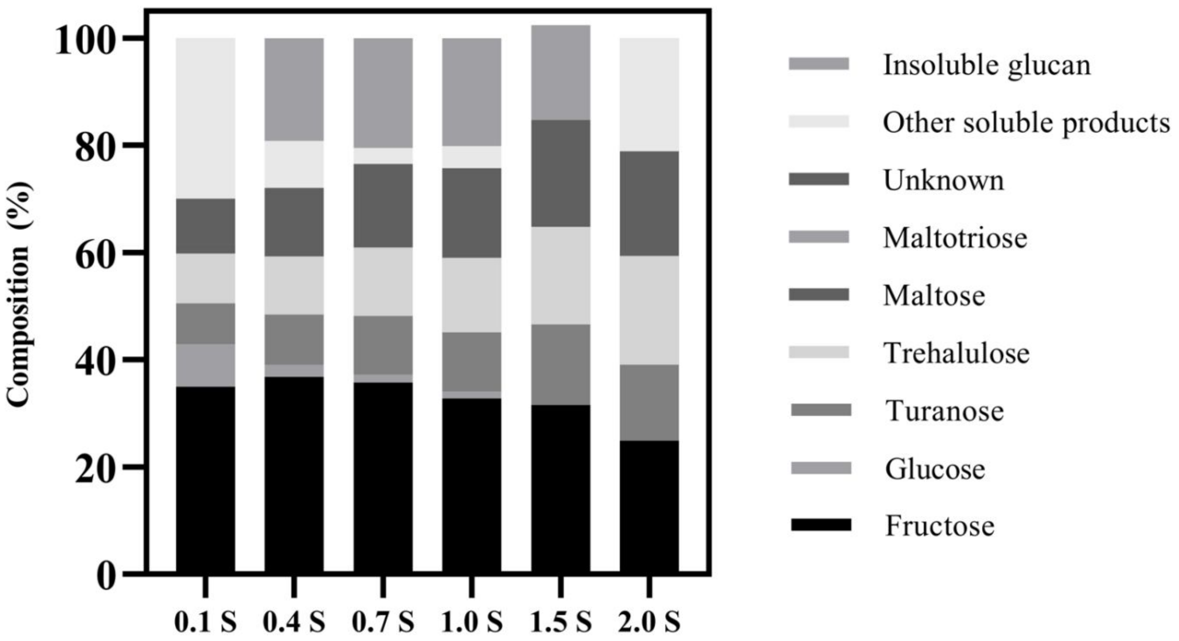

Figure S2. Composition of reaction products at various sucrose concentrations. The reactions were carried out using 400 U/L *Dd*AS enzyme in 50 mM sodium phosphate buffer (pH 7.5) at 35°C for 120 hr. Sucrose concentrations ranged from 0.1 S to 2.0 S, where S represents the molar concentration of sucrose (M).

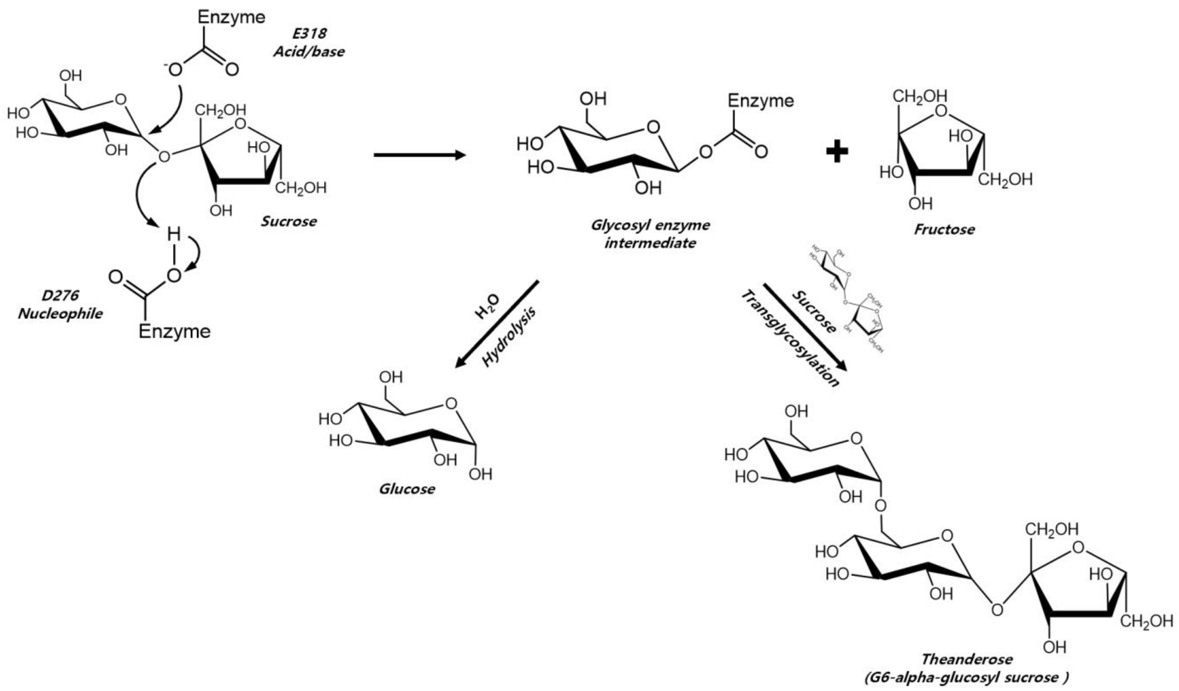

Figure S3. Mechanism of theanderose synthesis by amylosucrase. This figure illustrates the enzymatic process of sucrose breakdown and subsequent theanderose formation through transglycosylation.

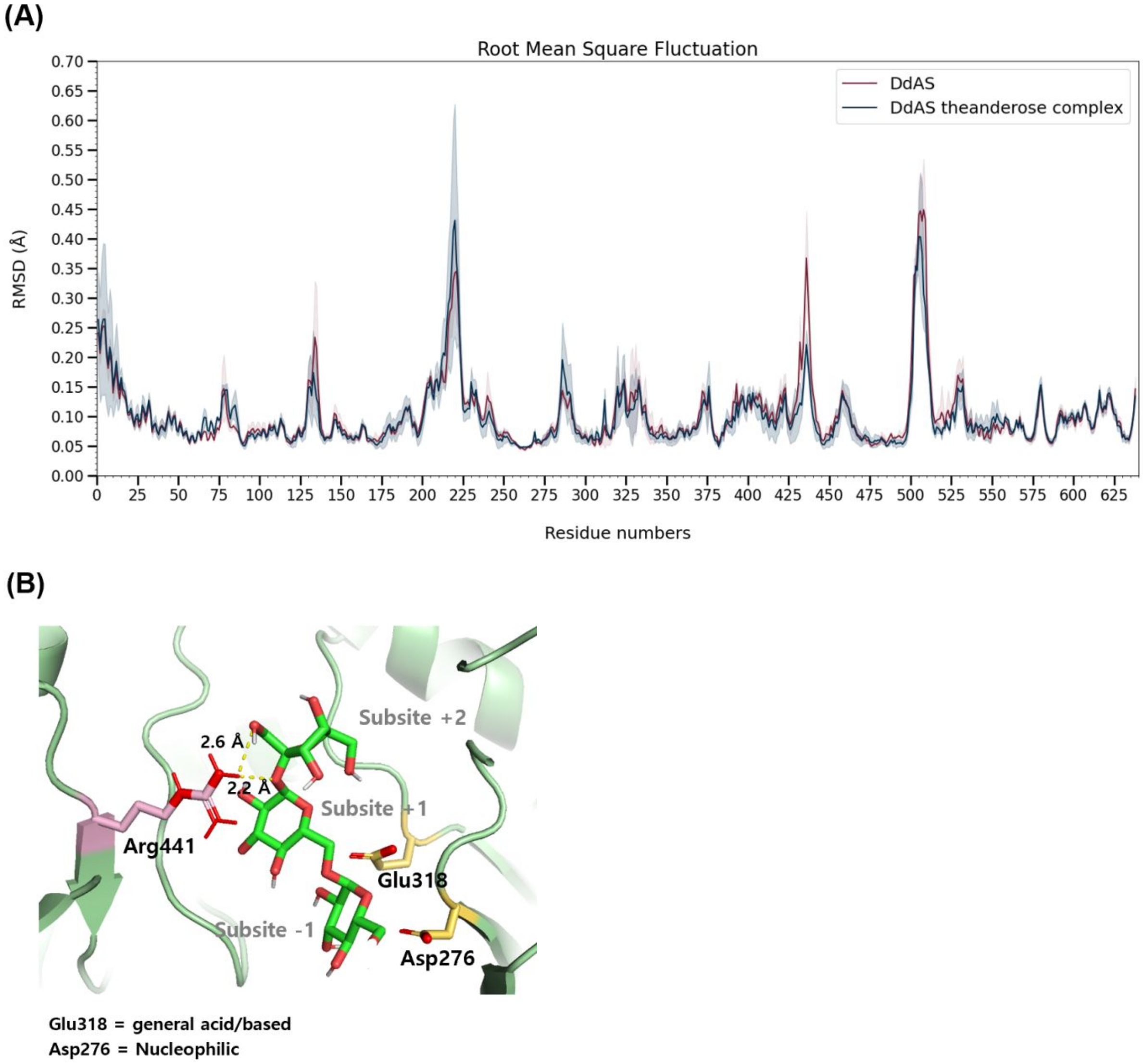

Figure S4. Molecular dynamics simulation results and binding site visualization of *Dd*AS complex with theanderose. (A) Root mean square fluctuation (RMSF) calculated from 3 independent molecular dynamics (MD) simulations for *Dd*AS (red) and *Dd*AS-theanderose complex (blue) (B) Visualization of the binding interaction between theanderose and key residues (Arg441, Glu318, Asp276) in the *Dd*AS, rendered using PyMOL. Subsites -1, +1, and +2 are indicated. Glu318 acts as a general acid/base catalyst, while Asp276 functions as a nucleophile.

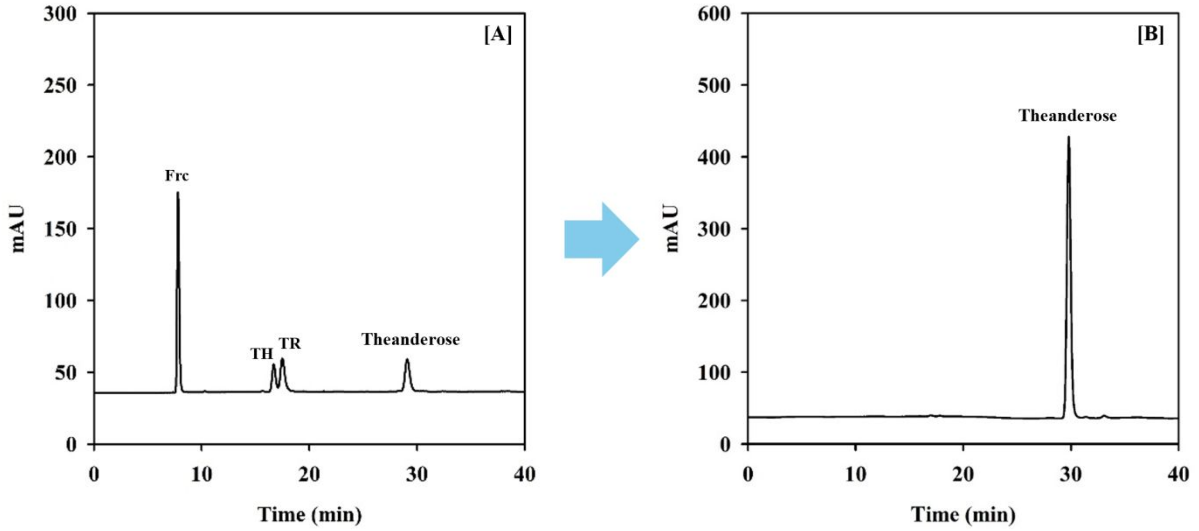

Figure S5. Targeted separation of *Dd*AS reaction product samples used for medium-pressure liquid chromatography (MPLC). Pre- [A] and post-separation [B] confirmed through high-performance liquid chromatography-evaporative light scattering detection (HPLC-ELSD).

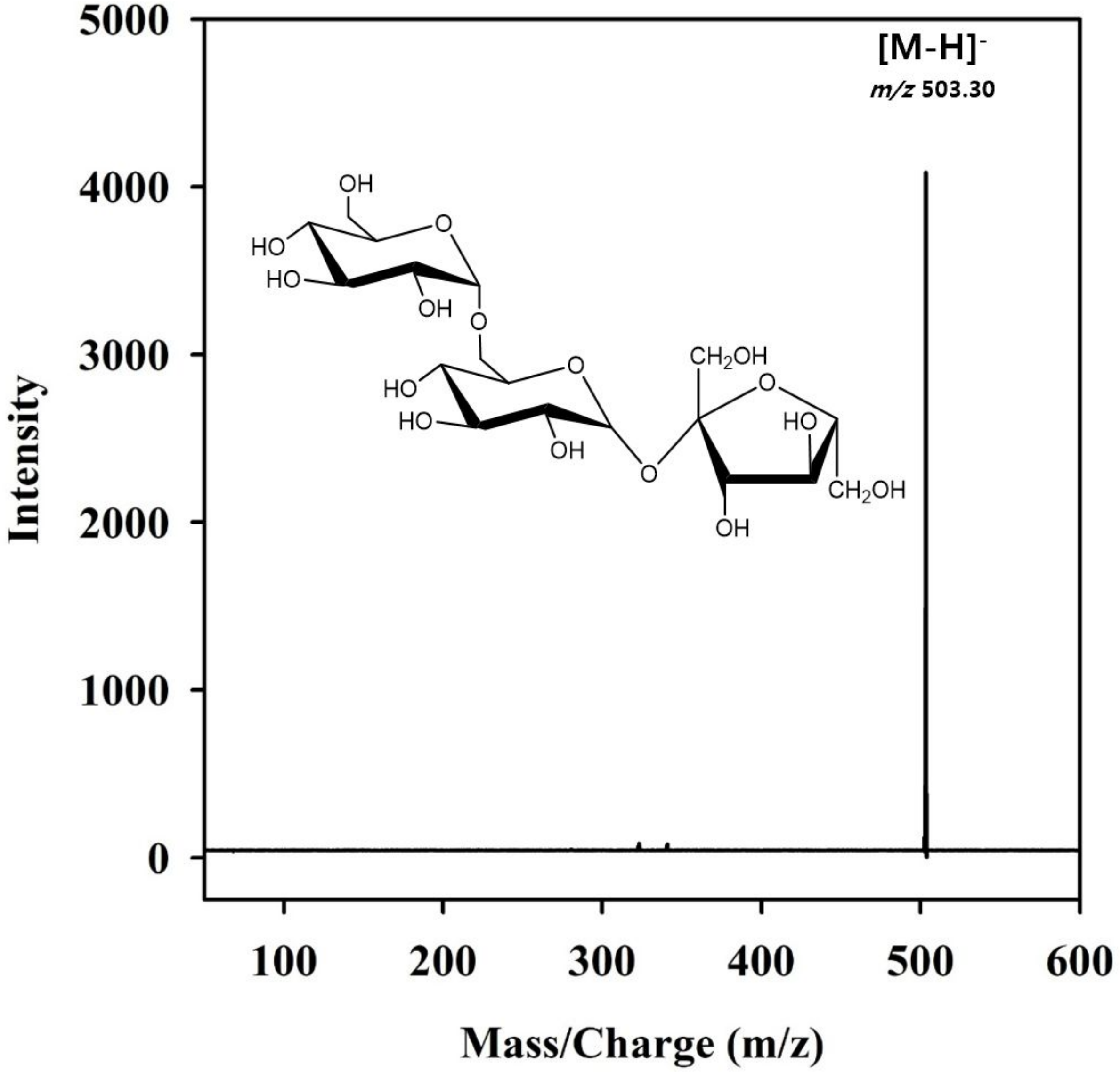

Figure S6. HPLC-MS chromatograms of purified theanderose: synthesis using *Dd*AS as 2.0 M sucrose substrate.

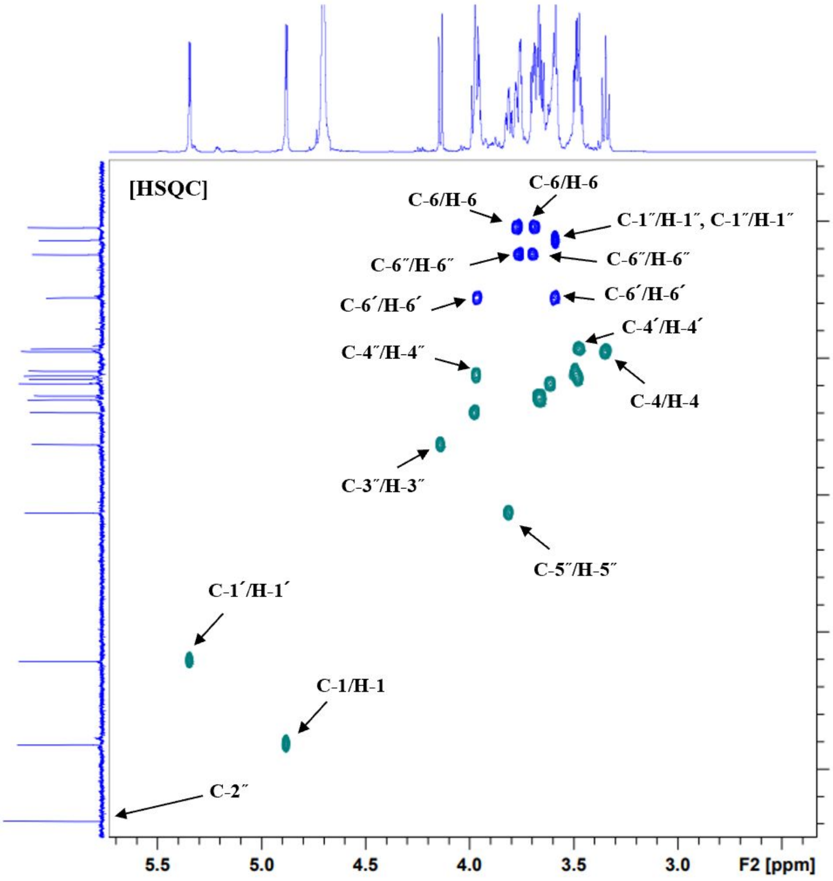

Figure S7. Heteronuclear Single Quantum Coherence (HSQC) of theanderose dissolved in D_2O.

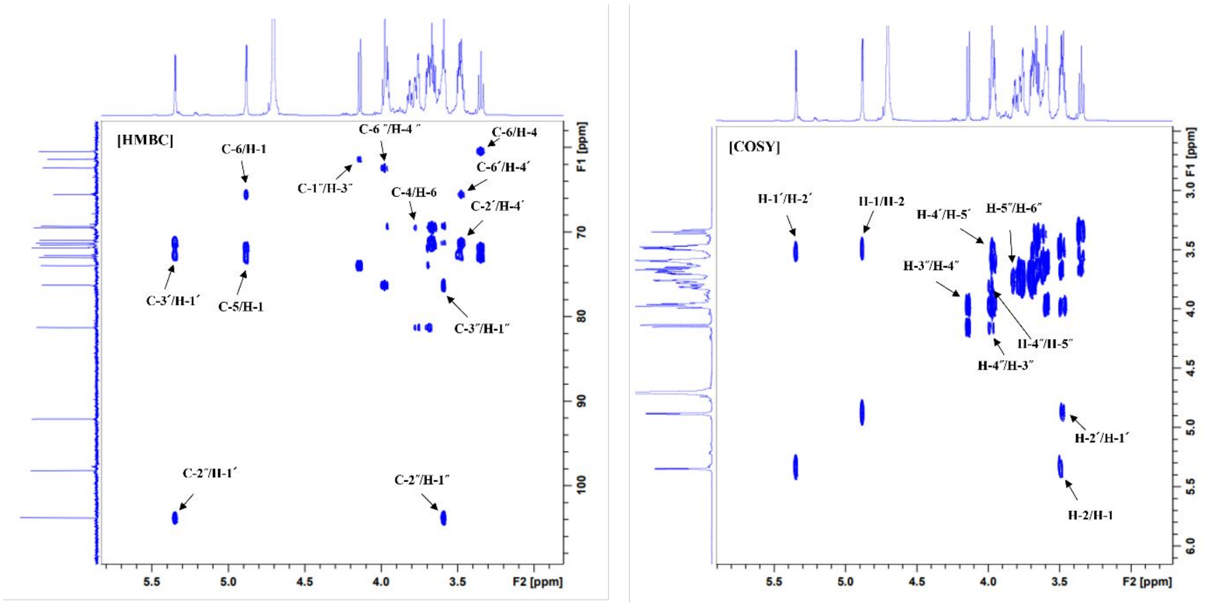

Figure S8. Heteronuclear Multiple Bond Correlation (HMBC) and Correlation Spectroscopy (COSY) of theanderose dissolved in D_2O.

Table S1. Growth of *Lactobacillus* & *Bifidobacterium* was verified using diverse carbon sources, with cultures incubated at 37℃ over a 24 h period. Glc, glucose; Thean, theanderose; Er, erlose; TR, turanose; TH, trehalulose; Raff, raffinose; G3, maltotriose. 30% or more growth over blank (+), 100% or more growth (++), 300% or more growth (+++)

Strains	**Blank**	**Glc**	**Thean**	**Er**	**TR**	**TH**	**Raff**	**G3**
LGG	-	+++	-	+	+	++	+	+
W.C	-	++	-	-	-	+	+	-
L.Para	-	++	-	-	-	-	-	-
L.casei	-	++	+	-	-	-	-	-
B.adolescentis	-	+++	+++	+++	+	+	++	+++
B.tsurumiense	-	+++	+++	+++	-	-	+++	+++
B.longum	-	+++	+++	++	+	+	+++	+

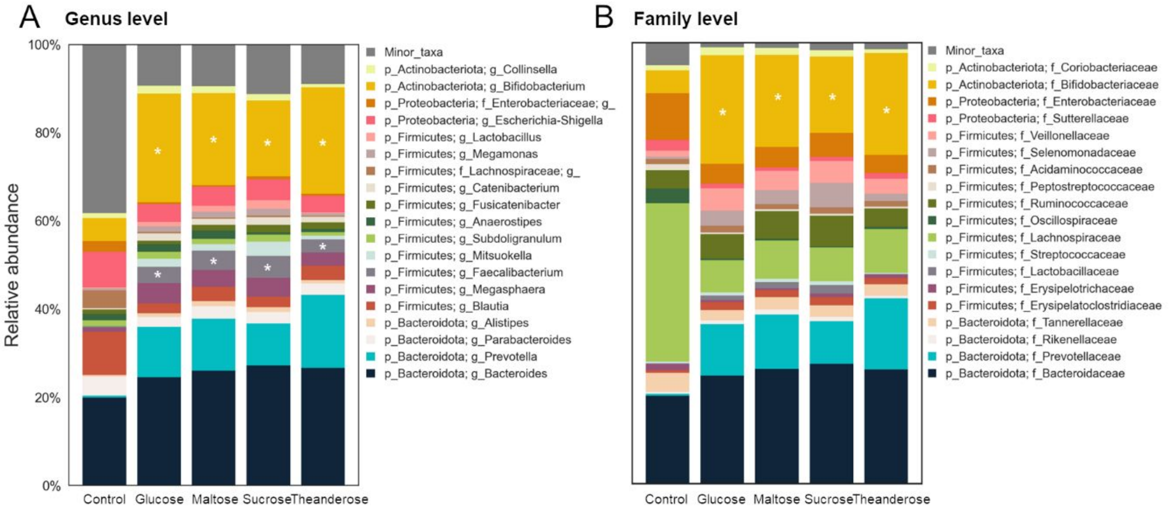

Figure S9. Changes in the gut microbiota composition at the (A) genus and (B) family level in response to treatments with glucose, maltose, sucrose, and theanderose. White stars indicate taxa differentially enriched by carbohydrate intervention.

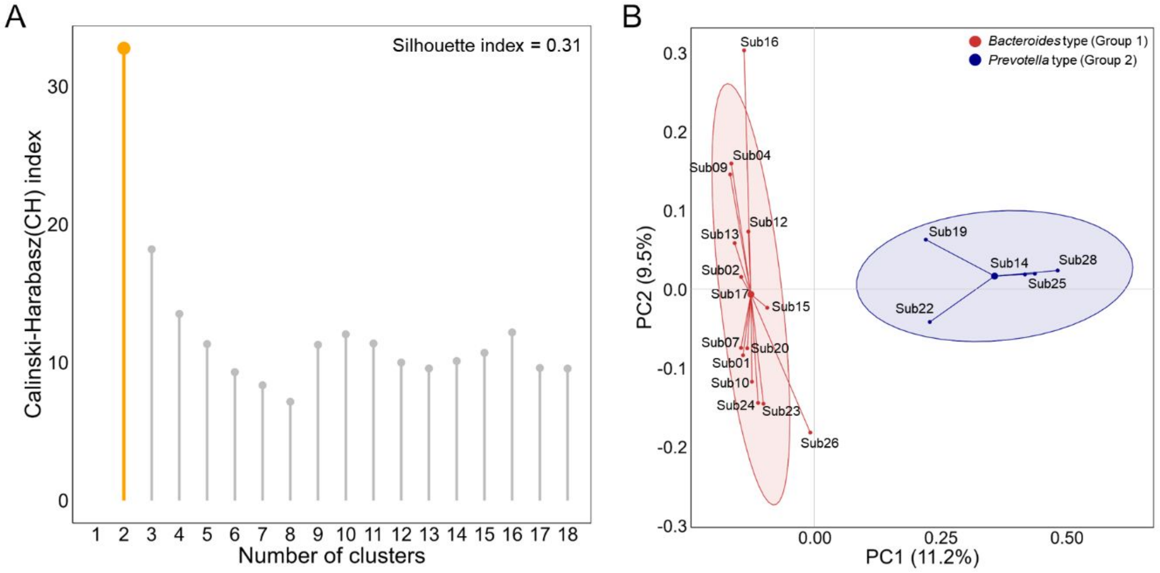

Figure S10. Gut enterotype classification of subjects. (A) Evaluated Calinski-Harabasz (CH) index for every number of k clusters to determine the optimal number of clusters. (B) Principal coordinates analysis (PCoA) plot of enterotyping data based on relative abundance of genus using the Jensen-Shannon divergence (JSD) distance matrix and the partitioning around medoids (PAM) clustering algorithm.

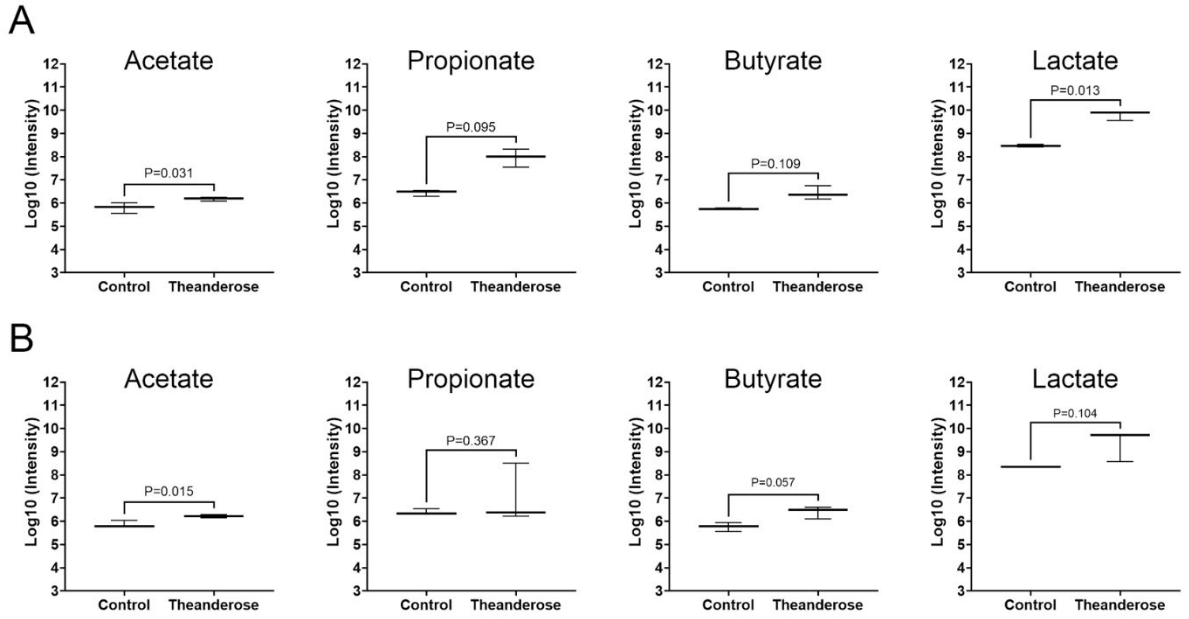

Figure S11. The comparison of amount of SCFAs between the control and theanderose-treated groups for *Bacteroides* (A) and *Prevotella* (B) types. Statistical significance (adjusted $P < 0.05$) was determined based on unpaired *t*-test.

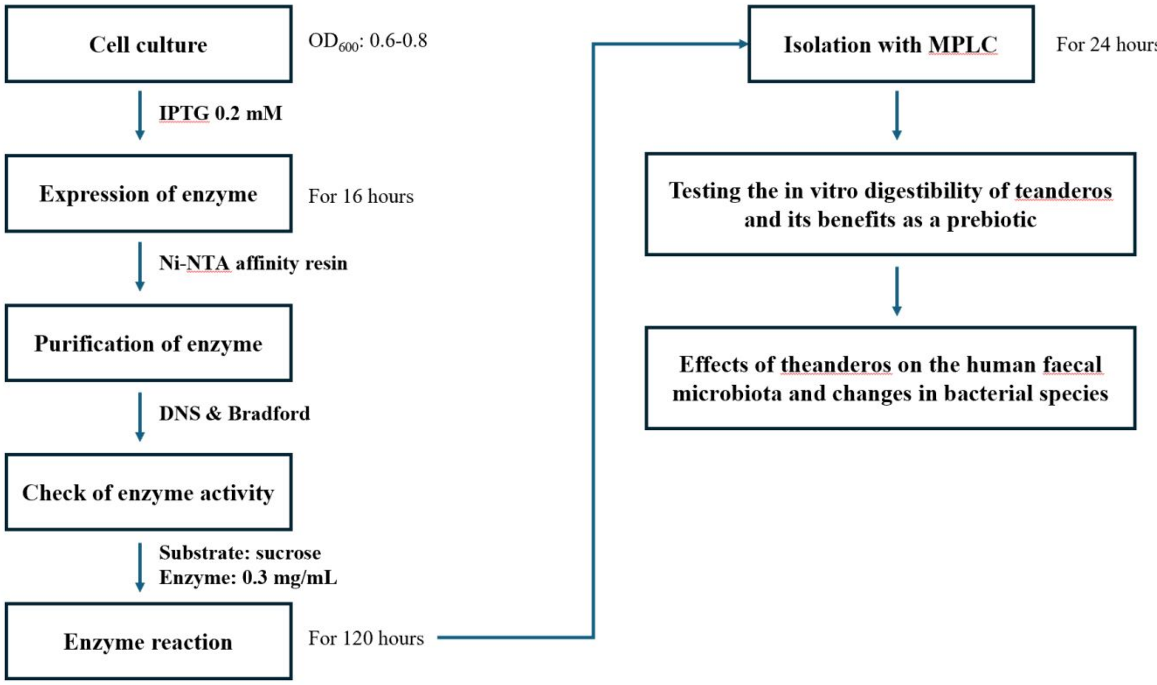

Figure S12. Flowchart of amylosucrase production, theanderose synthesis, and prebiotic effect evaluation.
